# The transcription factors AdNAC3 and AdMYB19 regulate kiwifruit ripening through brassinosteroid and ethylene signaling networks

**DOI:** 10.1093/plphys/kiaf084

**Published:** 2025-02-20

**Authors:** Yaming Yang, Ming Chen, Qinggang Zhu, Yanrong Lv, Cuihua Liu, Yun Wei, Guili Cha, Xiaoyan Shi, Xiaolin Ren, Yuduan Ding

**Affiliations:** College of Horticulture, Northwest A&F University, Yangling, Shaanxi 712100, China; College of Horticulture, Northwest A&F University, Yangling, Shaanxi 712100, China; College of Horticulture, Northwest A&F University, Yangling, Shaanxi 712100, China; College of Horticulture, Northwest A&F University, Yangling, Shaanxi 712100, China; College of Horticulture, Northwest A&F University, Yangling, Shaanxi 712100, China; College of Horticulture, Northwest A&F University, Yangling, Shaanxi 712100, China; College of Horticulture, Northwest A&F University, Yangling, Shaanxi 712100, China; College of Horticulture, Northwest A&F University, Yangling, Shaanxi 712100, China; College of Horticulture, Northwest A&F University, Yangling, Shaanxi 712100, China; College of Horticulture, Northwest A&F University, Yangling, Shaanxi 712100, China

## Abstract

The pivotal role of ethylene (ETH) in fruit ripening has been extensively studied; however, the function of brassinosteroids (BRs) in regulating fruit ripening remains poorly understood. Specifically, the mechanism by which BRs interact with ETH to affect kiwifruit (*Actinidia deliciosa*) ripening is unclear. Our research showed that 2 genes encoding transcription factors, *AdNAC3* and *AdMYB19*, and the fruit softening gene *AdEXP3* (encoding a cell wall expansion protein, expansin 3) were upregulated by ETH and downregulated by BRs. Furthermore, AdNAC3 and AdMYB19 positively regulated the activity of the *AdEXP3* promoter, and AdNAC3 positively regulated the promoter activity of *AdMYB19*. The physical interaction between AdNAC3 and the B-box-type zinc finger protein AdBBX32 affected fruit ripening. Transient overexpression and silencing experiments revealed that ETH upregulated and BRs downregulated the expression of *AdNAC3* and *AdMYB19*, thereby regulating the expression level of *AdEXP3* and participating in pectin degradation. Stable transformation of *AdNAC3* in tomato fruits accelerated fruit color change and promoted fruit ripening. These results indicate that AdNAC3 and AdMYB19 are involved in the hormone interaction between BRs and ETH in regulating kiwifruit ripening, providing insights into the molecular mechanisms underlying the crosstalk between BRs and ETH.

## Teaser

Transcription factors NAC3 and MYB19 interact to mediate the antagonistic effect of brassinosteroids on ethylene in regulating kiwifruit ripening.

## Introduction

Mature fruits play a crucial role in the human diet by providing a wealth of nutrients, such as sugars, organic acids, vitamins, minerals, and dietary fiber, which contribute substantially to human health and nutrition (Slavin and Lloyd 2012; Wang et al. 2023). The ripening process of fruits involves complex physiological and biochemical changes, including color changes, texture softening, the formation of aromatic compounds, and changes in flavor and texture (Giovannoni 2004; Li et al. 2010). These changes not only enhance the sensory quality of fruits but also influence their postharvest shelf life and market competitiveness. During postharvest processing and transport, fruits are prone to rapid ripening and softening, leading to quality deterioration and a shortened shelf life (Tucker et al. 2017). The high levels of respiration and ethylene (ETH) production, accompanied by increased activity of cell wall-degrading enzymes, lead to a decrease in fruit firmness and accelerate decay (Watkins 2006). Fruit softening, one of the key characteristics of ripening, is marked by significant changes in texture, primarily due to the degradation of cell wall components (Ding et al. 2015; Shi et al. 2022). During fruit ripening, a suite of cell wall-degrading enzymes, including polygalacturonase (PG), pectinesterase, pectate lyase (PL), xyloglucan endotransglucosylase/hydrolase (XTH), β-galacturonase, cellulase (Cel), expansins (Exp), and β-galactosidase, synergistically degrade pectin and cellulose in the cell wall, leading to fruit softening (Wei et al. 2018; Yang, Zhao, et al. 2024; Yang, Ren, et al. 2024). This coordinated enzymatic activity facilitates cell wall degradation and promotes the ripening of fruits.

Numerous studies have shown that the ripening of fleshy fruits is regulated by both genetic and environmental factors, with plant hormones playing a crucial role (Fang et al. 2024; You et al. 2024). ETH has been demonstrated to be closely associated with the ripening of various fruits at the physiological, biochemical, and molecular levels (Zhang et al. 2018; Wei, Yang, et al. 2023). In kiwifruits, tomatoes, apples, and bananas, ETH treatment significantly promotes fruit ripening, and treatment with 1-methylcyclopropene (1-MCP), an ETH inhibitor, notably delays fruit ripening (Zhang et al. 2018; Wang and Seymour 2022; Zhu et al. 2023; Wei, Liu, et al. 2023). Brassinosteroids (BRs), a class of plant steroid hormones, are involved in plant cell elongation, division, flower bud differentiation, and fruit development and ripening (Hong et al. 2018; Meng et al. 2023). Increasing evidence suggests that BR and other hormones also play significant roles in the fruit ripening process (Yang, Zhao, et al. 2024; Yang, Ren, et al. 2024). Exogenous BR treatment promotes the ripening of climacteric fruits such as tomatoes, bananas, mangoes, and persimmons and inhibits the ripening of apples, pears, and kiwifruits (Yang et al. 2023). In tomatoes, mangoes, and bananas, exogenous BR treatment stimulates the expression of genes related to ETH biosynthesis, increasing ETH production and thereby accelerating fruit ripening (Zaharah et al. 2012; Zhu et al. 2015; Guo et al. 2019). By contrast, in apples and pears, exogenous BR treatment delays the decrease in fruit firmness and the ETH production, delaying the ripening process (Ji et al. 2021). All of these results suggest that BR influences fruit ripening through interactions with ETH, but the underlying mechanisms remain to be elucidated.

During fruit ripening and softening, the crosstalk between plant hormones forms a complex regulatory network that coordinates physiological and molecular changes (Ding et al. 2014; Kumar et al. 2014). ETH, as the primary ripening hormone, interacts with other hormones such as auxin (Aux), BRs, salicylic acid (SA), jasmonic acid (JA), and abscisic acid (ABA), influencing fruit ripening and texture changes. For example, in tomatoes, Aux delays ripening by inhibiting ETH synthesis. In kiwifruits, apples, and pears, BRs inhibit ETH synthesis and signaling transduction, which delays fruit ripening (Lu et al. 2019; Ji et al. 2021). In apricots, SA prolonged storage by inhibiting ETH biosynthesis and cell wall-degrading enzyme activity (Li et al. 2022). In addition, studies have shown that ETH crosstalks with JA and ABA, playing a role in the ripening of peach and tomato fruits (Ziosi et al. 2008; Zhang et al. 2009). Transcription factors (TFs) function as key regulatory hubs in the plant hormone signaling network, integrating the crosstalk between different hormone pathways and regulating fruit ripening. In papayas, CpARF2 (auxin response factors) physically interacts with CpEIL1 (Ethylene Insensitive-Like) to regulate Aux and ETH signaling (Zhang et al. 2020). In apples, MdMYC2 coordinates the synergistic crosstalk between JA and ETH signaling by regulating *MdERF3* (ethylene response factor) and ETH biosynthesis genes involved in ETH biosynthesis (Li et al. 2017). However, in the ripening process of kiwifruit, although both ETH and BR have been shown to be involved in the regulation of ripening, the crosstalk between ETH and BR and its specific mechanisms on kiwifruit ripening have not yet been elucidated. Considering the pivotal role of TFs in hormone signaling crosstalk, the regulatory mechanisms of key TFs during the crosstalk between ETH and BR in kiwifruit ripening and softening warrant further exploration.

Kiwifruit is an essential economic fruit crop and a typical climacteric fruit, characterized by a dramatic increase in ETH production during the late ripening stage, making it highly sensitive to ETH (McDonald and Harman 1982). Previous studies have shown that various TFs are involved in the regulatory network regulating kiwifruit ripening and softening. For instance, the TFs AdEIL2 and AdEIL3 have been shown to activate the expression of ripening-related genes *AdACO1* and *AdXET5*, which promote fruit ripening (Yin et al. 2010). AcWRKY40 activates the expression of ETH biosynthesis genes *AcSAM2*, *AcACS1*, and *AcACS2*, contributing to postharvest ripening (Gan, Yuan, Shan, Wan, Chen, Xu, et al. 2021). The zinc finger TF AdZAT5 promotes fruit ripening by activating the expression of *AdPL5* and *Adβ-Gal5*, which enhance pectin degradation (Zhang et al. 2022). AdNAC2 and AdNAC72 directly bind to the *AdMsrB1* (a methionine sulfoxide reductase gene) promoter that regulates its expression and promotes ETH production (Fu, Wang, et al. 2021). However, fruit ripening and softening are complex biological processes involving various TFs and their interplay. Exp is a key regulator of cell wall loosening and fruit softening, and its expression is regulated by multiple TFs (Cosgrove 2000). Studies have shown that the NAC and MYB TF families play significant roles in regulating fruit-ripening-related genes (Yang, Chen, et al. 2022; Li, Yan, et al. 2023). Furthermore, the B-box-type zinc finger protein (BBX) TF family, which is an essential regulator of light signal transduction and growth development, may interact with other TFs to coregulate the expression of target genes (Gangappa and Botto 2014). A deeper understanding of the functions of these TFs and their interactions will help in elucidating the molecular mechanisms underlying kiwifruit ripening.

In this study, we explored the crosstalk between BR and ETH during kiwifruit ripening. On the basis of the physiological experiments and transcriptomic analysis, we identified key TFs involved in the ripening process of kiwifruit. The AdNAC3 and AdMYB19 directly activate the expression of ripening-related genes, with ETH upregulating and BR downregulating their expression. This study suggests that AdNAC3 and AdMYB19 are participants in BR and ETH signaling and in regulating fruit ripening, which provides insights into the regulatory network of BR and ETH in fruit ripening.

## Results

### BR and ETH treatments influenced postharvest ripening of kiwifruit

**Figure 1.**
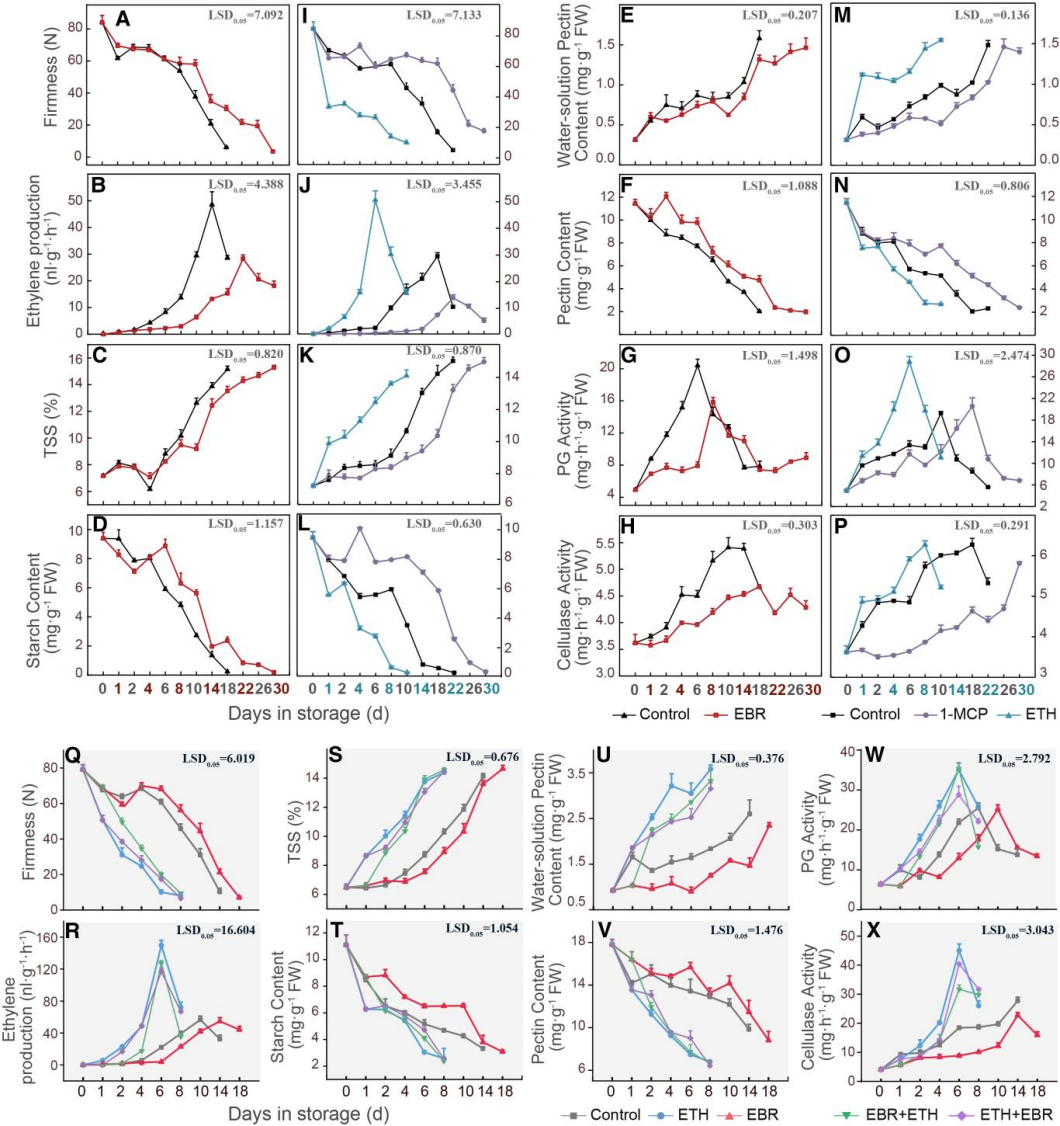
Effects of ETH (100 *μ*L/L), ETH inhibitor 1-MCP (1 *μ*L/L), and BR analog EBR (10 *μ*m), as well as the combination of ETH and EBR, on the ripening of ‘Hayward’ kiwifruit. The impact of EBR, ETH, 1-MCP, and control treatments on **A, I, Q)** fruit firmness, **B, J, R)** ETH production, **C, K, S)** TSS content, **D, L, T)** starch content, **E, M, U)** WSP, **F, N, V)** pectin, **G, O, W)** PG activity, and **H, P, X)** Cel activity. The error bars for firmness and TSS represent Se based on 12 biological replicates. For ETH production, TSS content, starch content, WSP, pectin, PG activity, and Cel activity, the error bars represent Se calculated from 3 biological replicates. Lsd indicates significant differences as determined by the Lsd test (*P* = 0.05). FW, fresh weight of the fruit.

To explore the role of BR in postharvest ripening of kiwifruit, 2,4-epibrassinolide (EBR) treatment was applied, which significantly delayed fruit ripening. In the EBR-treated group, fruit firmness decreased to 3.4 N on Day 34, whereas the control group showed a reduction to 5.9 N on Day 22 (Fig. 1A). Compared with the control, EBR treatment reduced the peak of ETH production (Fig. 1B), inhibited the accumulation of total soluble solids (TSS) (Fig. 1C) and starch degradation (Fig. 1D), and enhanced the accumulation of endogenous BR content in fruits (Supplementary Fig. S1). Moreover, exogenous EBR treatment significantly enhanced the accumulation of water-soluble pectin (WSP) (Fig. 1E) and delayed pectin degradation (Fig. 1F). Similarly, EBR treatment reduced the activity of cell wall-degrading enzymes, such as and PG (Fig. 1G) and Cel (Fig. 1H). These results suggest that BR can delay the postharvest ripening of kiwifruit.

Kiwifruit is a typical climacteric fruit, and ETH treatment significantly promotes fruit ripening, whereas 1-MCP treatment markedly delays the ripening process. ETH-treated fruits showed a rapid decrease in firmness (Fig. 1I), an earlier peak in ETH production (Fig. 1J), and a swift reduction in starch content (Fig. 1L), which potentially facilitated the accumulation of TSS (Fig. 1K). ETH treatment also induced a significant enhancement in the cell wall-degrading enzyme activities of PG (Fig. 1O) and Cel (Fig. 1P), which facilitated pectin degradation (Fig. 1N) and the accumulation of WSP (Fig. 1M). By contrast, 1-MCP-treated fruits exhibited the opposite effects. These results indicate that ETH plays a key role in promoting the ripening of kiwifruit.

To further elucidate the role of the crosstalk between BR and ETH in kiwifruit ripening, a combination of ETH and BR treatments was applied to assess changes in fruit ripening phenotypes. EBR treatment was applied first, followed by ETH treatment (EBR + ETH), the decrease in fruit firmness was significantly lower than that with ETH treatment alone. ETH treatment was applied first, followed by EBR treatment (ETH + EBR), the change in firmness was intermediate between the ETH-only treatment and EBR + ETH treatment (Fig. 1Q). In addition, we measured ETH production (Fig. 1R), TSS (Fig. 1S), starch content (Fig. 1T), pectin content (Fig. 1, U and V), changes in PG, (Fig. 1W), and Cel enzyme activities (Fig. 1X), which were consistent with the fruit softening phenotype. These results further indicate that ETH treatment accelerates kiwifruit ripening and BR treatment delays ripening. However, in the combined BR and ETH treatment, the presence of BR partially attenuates the ETH-induced acceleration of ripening.

### RNA-seq analysis and identification of key TFs and structural genes during kiwifruit ripening

**Figure 2.**
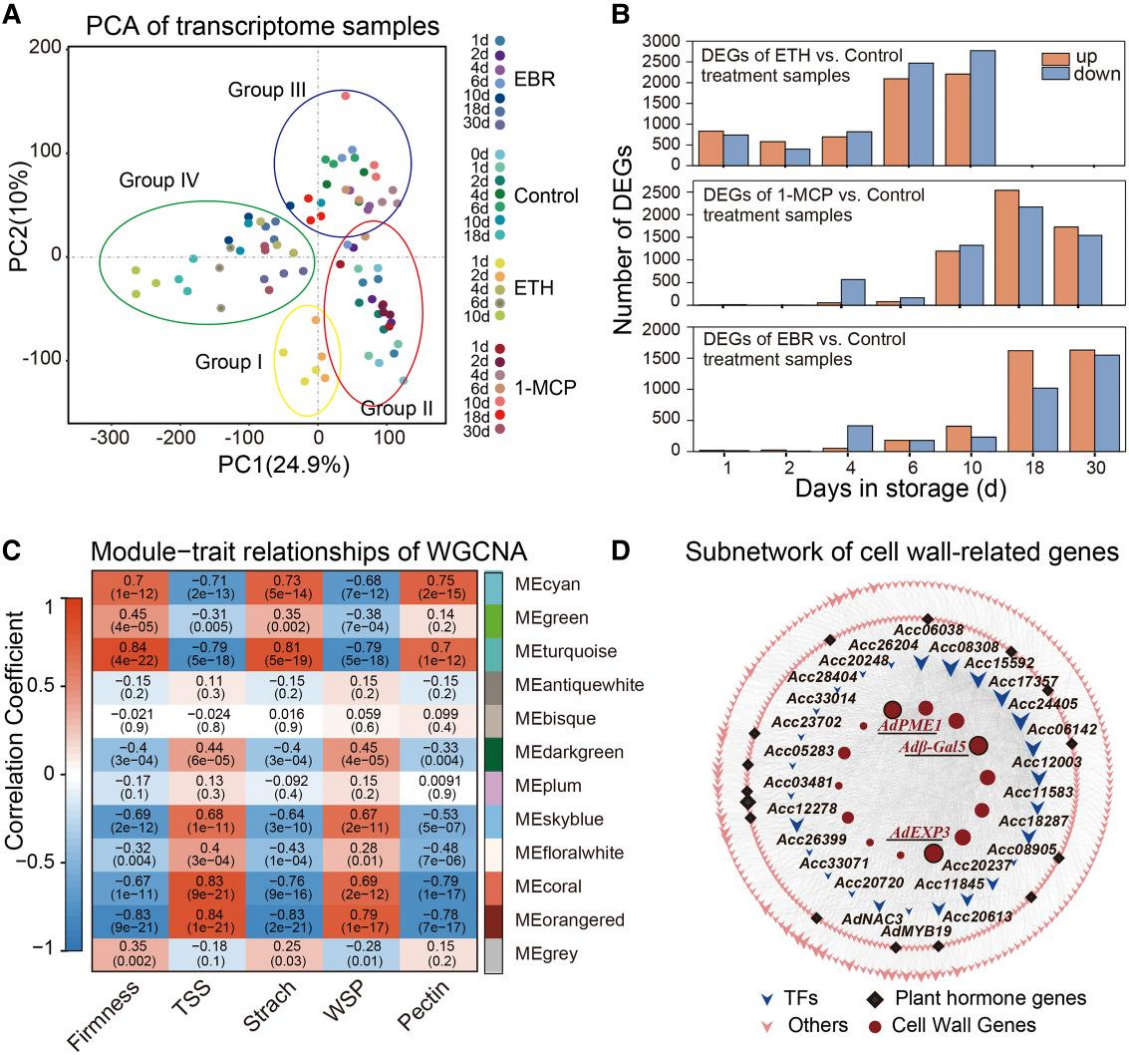
RNA-seq analysis and WGCNA identification of key genes among different treatments. **A)** PCA of kiwifruit samples at each time point after treatment, based on 3 biological replicates. **B)** Number of DEGs at each time point under different treatments compared to the control, based on 3 biological replicates. **C)** Correlation between gene modules and fruit softening. **D)** Subnetwork of cell wall-related genes constructed in the “MEorangered” module. 1-MCP, 1-methylcyclopropene, an ETH receptor antagonist; EBR, brassinosteroid analog 2,4-epibrassinolide; ETH, ethylene.

Large-scale RNA-sequencing (RNA-seq) analysis further revealed the dynamic changes in genes involved in fruit ripening under ETH and EBR treatments. To accurately assess the time-dependent variations of gene expression, RNA-seq was performed at key time points for the control, ETH, 1-MCP, and EBR treatments in 2021 at 8 time points (0, 1, 2, 4, 6, 10, 18, and 30 d), with a total of 78 samples (Supplementary Table S1). To assess the relationships between biological replicates and determine sample variations, principal component analysis (PCA) was performed. PCA focusing on differentially expressed genes (DEGs) revealed that the 3 biological replicates for each time point clustered tightly. Although minor deviations due to sampling variability were observed, the overall clustering indicated acceptable consistency across samples at each time point (Fig. 2A). Importantly, the PCA score plot also revealed significant differences between time points. Under different treatments, the DEGs were mainly concentrated in the later stages of fruit ripening (Fig. 2B). Gene ontology enrichment analysis indicated that DEGs under different treatments were primarily enriched in biological processes related to cell wall organization (Supplementary Fig. S2, A, C, and E). Kyoto Encyclopedia of Genes and Genomes pathway analysis showed that DEGs were primarily enriched in pathways related to plant hormone signal transduction and carbon metabolism (Supplementary Fig. S2, B, D, and F).

To further identify genes coinduced by BR and ETH, we performed a combined analysis of transcriptome data and phenotypic traits (including firmness, TSS content, starch content, WSP, and pectin content) and developed a weighted gene coexpression network (WGCNA). Genes with similar expression patterns were clustered into the same modules (Supplementary Fig. S3A). Following their expression profiles, these genes were categorized into 12 modules, with the genes in the ‘MEorangered’ module showing the highest correlation with phenotypic traits, and were therefore prioritized for further analysis (Fig. 2C). Cell wall degradation plays a critical role in the process of fruit softening. Thus, we focused on 14 genes involved in cell wall softening in the ‘MEorangered’ module. To further identify key genes and regulatory networks among these 14 cell wall degradation genes, we constructed a regulatory subnet of the 14 cell wall degradation genes in the ‘MEorangered’ module. In this network, 3 cell wall degradation genes (*AdPME1*, *AdEXP3*, and *Adβ-Gal5*) with a high number of connections and 21 TFs potentially regulating these genes were prioritized (Fig. 2D). Correlation analysis revealed a strong positive correlation between these 21 TFs and the 3 cell wall degradation genes (Supplementary Fig. S3B). Of these 21 TFs, 19 were successfully cloned for further analysis.

### Regulatory effects of key TFs on the promoters of 3 cell wall degradation genes and RT-qPCR analysis

**Figure 3.**
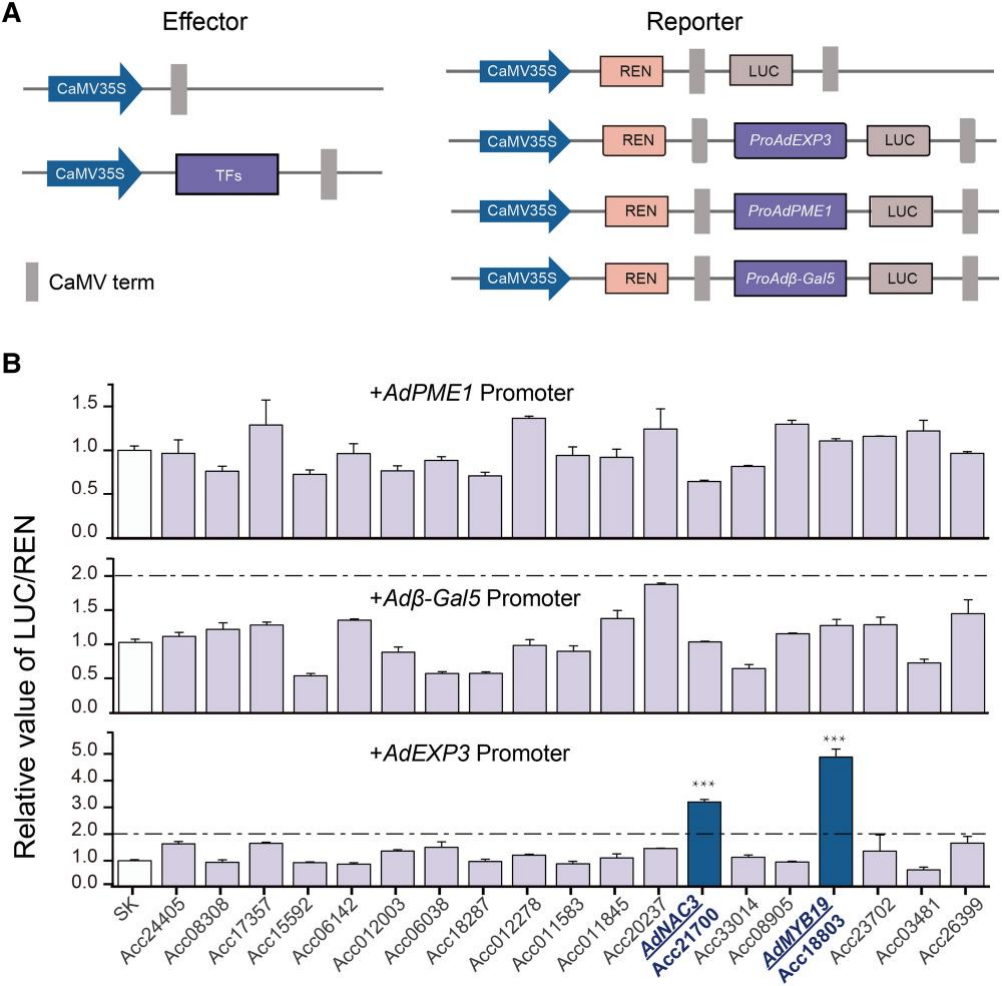
Dual-luciferase reporter assay to validate the regulatory effects of TFs on cell wall degradation genes. **A)** Schematic representation of the TFs and cell wall degradation genes. **B)** Regulatory activity of TFs on the promoters of *AdPME1*, *Adβ-Gal5*, and *AdEXP3*. The empty vector pGreen II002962-SK (SK) mixed with the promoter was used as a reference, with the LUC/REN ratio set to 1. Error bars represent Se based on 4 biological replicates. The dashed line indicates the 2-fold difference. Asterisks indicate significant differences by Student's *t*-test (***P < 0.001).

To validate the transcriptional regulatory effects of 19 TFs on 3 cell wall degradation genes (*AdPME1*, *AdEXP3*, and *Adβ-Gal5*), we performed the dual-luciferase reporter assay. Initially, we assessed the responses of the *AdPME1*, *AdEXP3*, and *Adβ-Gal5* promoters to ETH and BR in *Nicotiana benthamiana* leaves. After 6 and 12 h of treatment with ETH and EBR, no significant differences were observed in the promoter activity compared with the control group (Supplementary Fig. S4). These results suggest that ETH or EBR treatment does not directly modulate the *AdPME1*, *AdEXP3*, and *Adβ-Gal5* promoters in the *N. benthamiana* system. Further dual-luciferase assays revealed that AdNAC3 and AdMYB19 exhibited significant positive regulatory effects on the *AdEXP3* promoter, with fold changes (FCs) of 3.2 and 4.9, respectively (Fig. 3, A and B). However, no significant regulatory effects of other TFs on the *AdPME1* and *Adβ-Gal5* promoters were observed (Fig. 3B).

**Figure 4.**
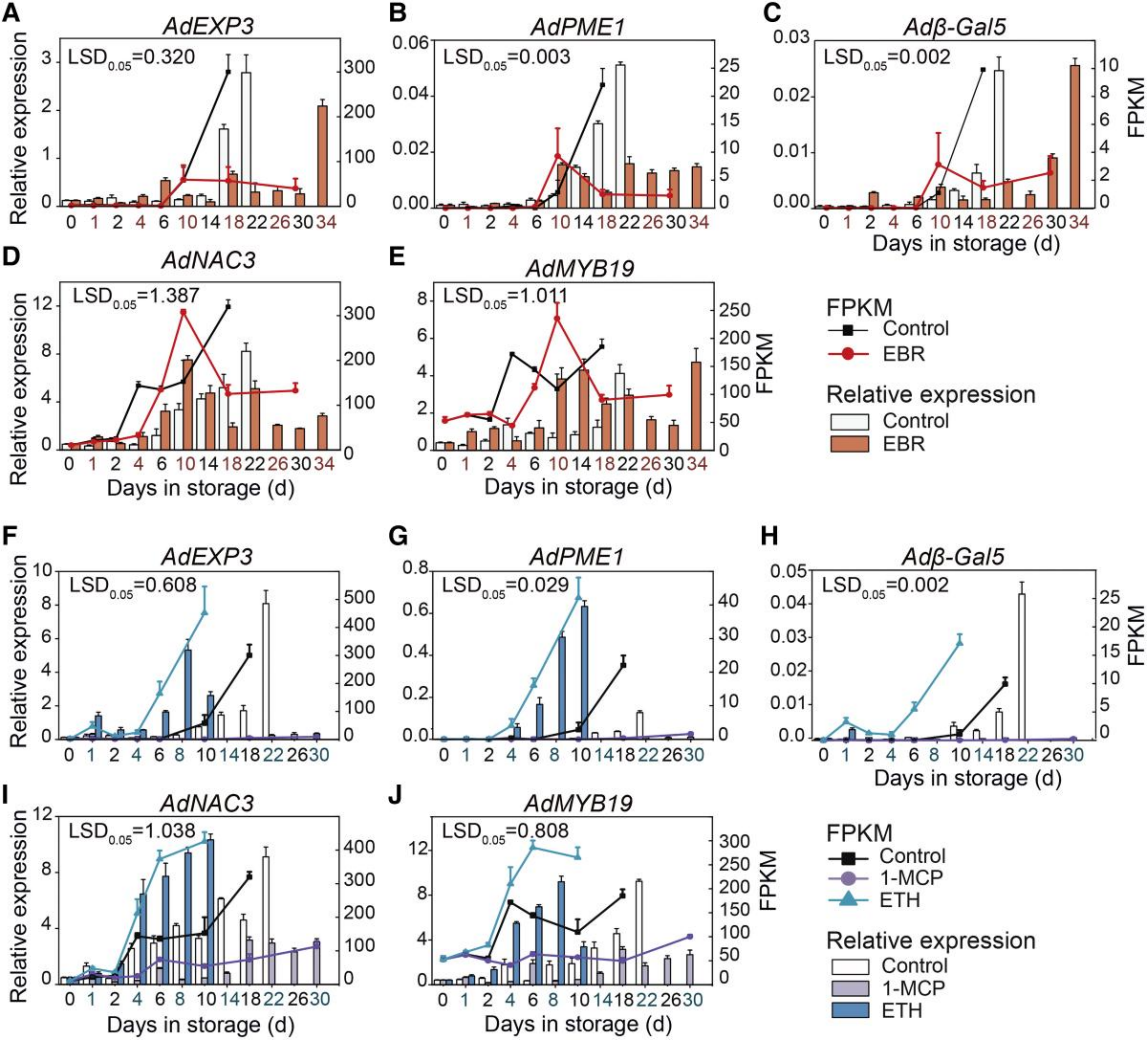
Gene expression levels during the ripening of ‘Hayward’ kiwifruit under different treatments. Transcriptome FPKM and RT-qPCR analyses of 3 cell wall degradation genes **A, F)**  *AdEXP3*, **B, G)**  *AdPME1*, and **C, H)**  *Adβ-Gal5* and 2 TFs **D, I)**  *AdNAC3* and **E, J)**  *AdMYB19* during fruit ripening under treatments with EBR, ETH, 1-MCP, and control. Error bars represent Se based on 3 biological replicates, and Lsd indicates significant differences (*P* = 0.05). 1-MCP, 1-methylcyclopropene, an ETH receptor antagonist; EBR, brassinosteroid analog 2,4-epibrassinolide; ETH, ethylene.

The expression patterns of 19 ripening-related TFs and 3 cell wall degradation genes under ETH and EBR treatments were analyzed using reverse transcription quantitative PCR (RT-qPCR). The expression patterns of the functional genes *AdEXP3*, *AdPME1*, and *Adβ-Gal5* were downregulated by EBR treatment (Fig. 4, A to C). In addition, the expression patterns were upregulated by ETH and downregulated by 1-MCP treatment (Fig. 4, F to H). The *AdNAC3* and *AdMYB19* exhibited similar expression patterns (Fig. 4, D, E, I, and J). In addition, *AdBZR1.1* (*Acc12003*) was included in our previous study (Yang, Zhao, et al. 2024; Yang, Ren, et al. 2024). The expression patterns of the other 16 TFs showed similar trends and were consistent with the RNA-seq (Supplementary Figs. S5 and S6). The RT-qPCR results of the combined ETH and EBR treatment further indicated that the expression patterns of *AdPME1*, *AdEXP3*, *Adβ-Gal5*, *AdNAC3*, and *AdMYB19* were strongly upregulated by ETH, with slightly lower expression levels in the combined ETH and BR treatment compared with the ETH-only treatment (Supplementary Fig. S7). These findings suggest that the expression patterns of *AdPME1*, *AdEXP3*, *Adβ-Gal5*, *AdNAC3*, and *AdMYB19* were jointly affected by ETH and BR treatments.

### Regulatory effects of TFs AdNAC3 and AdMYB19 on the promoter of *AdEXP3*

**Figure 5.**
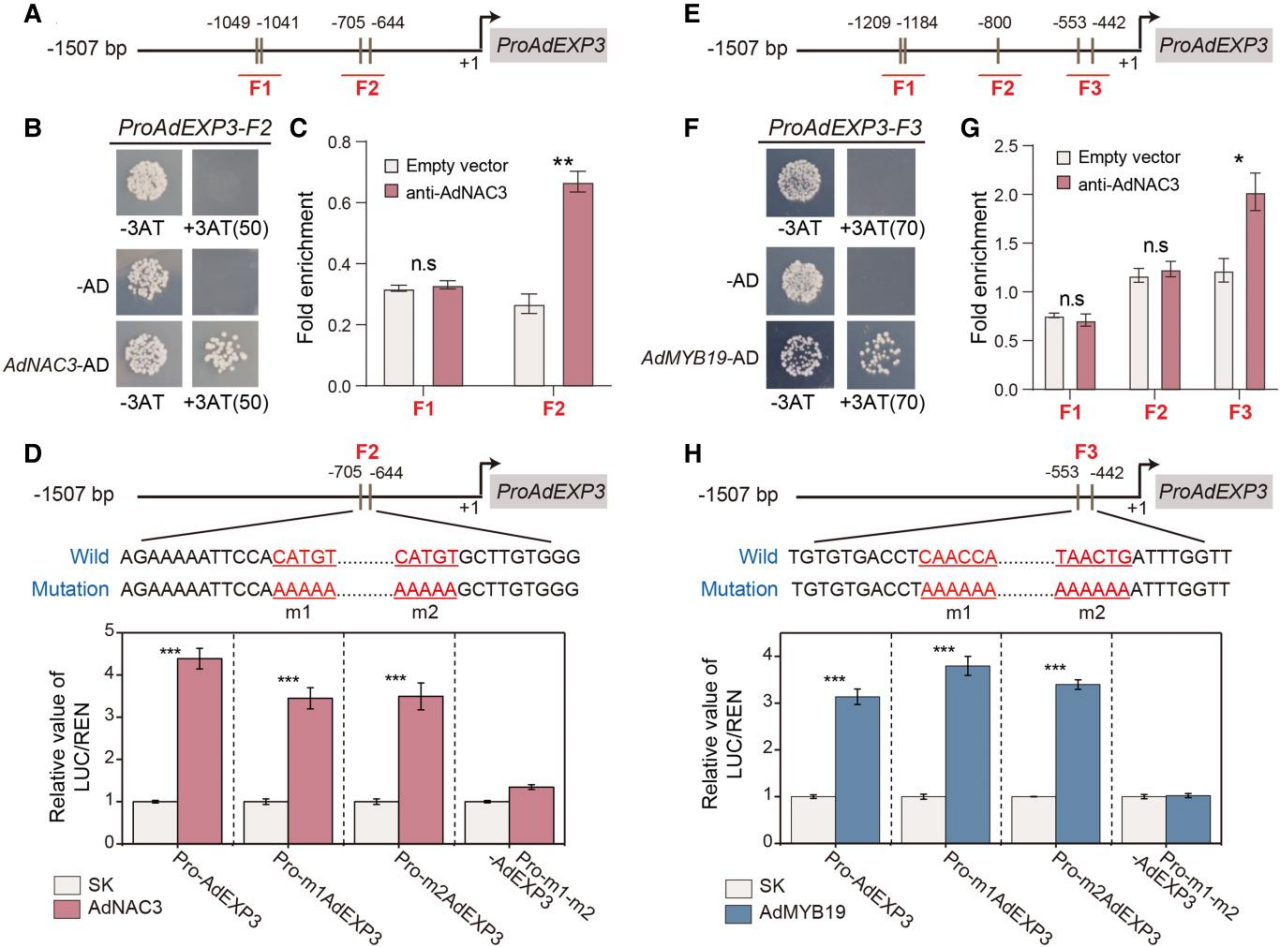
Regulatory effects of AdNAC3 and AdMYB19 on *AdEXP3*. **A)** Schematic representation of potential AdNAC3 binding sites within the *AdEXP3* promoter sequence. **B)** Y1H assay validating the binding of AdNAC3 on the *AdEXP3* promoter. The empty vector and the F2 fragment of the *AdEXP3* promoter were used as negative controls. Sd media lacking Trp, Leu, and His was supplemented with 50 mm 3-AT to further exclude the self-activation effect of the promoter. **C)** ChIP-qPCR analysis comparing the binding affinity of the anti-AdNAC3 GFP antibody to specific regions of the *AdEXP3* promoter. The empty vector (35S::GFP) served as a negative control for binding affinity. Error bars represent Se based on 3 biological replicates. **D)** Site-directed mutagenesis analysis to validate the binding effect of AdNAC3 on the *AdEXP3* promoter. m1 represents the first mutated position of the promoter, m2 represents the second mutated position, and -m1-m2 represents simultaneous mutation at both positions. Error bars represent Se based on 3 biological replicates. The WT promoter sequence was used as a control. Similarly, regulatory effect of AdMYB19 on *AdEXP3*, as analyzed in **E)** schematic of potential AdMYB19 binding sites in the *AdEXP3* promoter sequence, **F)** Y1H assay, **G)** ChIP-qPCR, and **H)** site-directed mutagenesis experiments to further validate this regulatory effect. The error bars for ChIP-qPCR and site-directed mutagenesis experiments represent Se based on 3 biological replicates. Asterisks indicate significant differences determined by Student's *t*-test (*P < 0.05, **P < 0.01, and ***P < 0.001); n.s. indicates no significant difference.

Considering that the dual-luciferase assays indicate AdNAC3 and AdMYB19 positively activate the activity of the *AdEXP3* promoter. To further confirm this regulatory effect, yeast 1-hybrid (Y1H) assays first confirmed that AdNAC3 bound specifically to the F2 fragment of the *AdEXP3* promoter (Fig. 5, A and B) whereas AdMYB19 bound to the F3 fragment of the *AdEXP3* promoter (Fig. 5, E and F). In addition, chromatin immunoprecipitation followed by qPCR (ChIP-qPCR) analysis provided further evidence that AdNAC3 and AdMYB19 showed marked enrichment at the F2 and F3 fragments of the *AdEXP3* promoter, indicating their direct regulatory role in *AdEXP3* promoter activity (Fig. 5, C and G). Finally, through site-directed mutagenesis, we demonstrated that AdNAC3 specifically bound to 2 CATGT motifs in the F2 region of the *AdEXP3* promoter (Fig. 5D) and AdMYB19 specifically bound to the CAACCA and TAACTG sequences in the F3 region of the same promoter, providing functional validation of their transcriptional regulatory effects (Fig. 5H). These results further support the direct regulatory role of AdNAC3 and AdMYB19 in the *AdEXP3* promoter and provide further evidence that AdNAC3 and AdMYB19 directly bind to the *AdEXP3* promoter. Furthermore, subcellular localization analysis showed that the transient expression of AdNAC3-GFP and AdMYB19-GFP in *N. benthamiana* leaves resulted in both proteins displaying green fluorescence in the nucleus, which colocalized with the red fluorescence of the mCherry nuclear marker, suggesting that AdNAC3 and AdMYB19, like most TFs, were localized in the nucleus (Supplementary Fig. S8).

Considering that both AdNAC3 and AdMYB19 positively regulate the expression of *AdEXP3*, we further explored the function of AdEXP3 by performing transient overexpression (OE) and silencing of *AdEXP3* in ‘Hayward’ kiwifruit. The transient OE experiment showed that the OE of *AdEXP3* significantly upregulated its expression and facilitated pectin and starch degradation. Conversely, silencing *AdEXP3* delayed pectin and starch degradation (Supplementary Fig. S9). These results further suggest that AdEXP3 plays a positive role in kiwifruit ripening and softening.

### Transcriptional regulation of the *AdMYB19* promoter by AdNAC3

**Figure 6.**
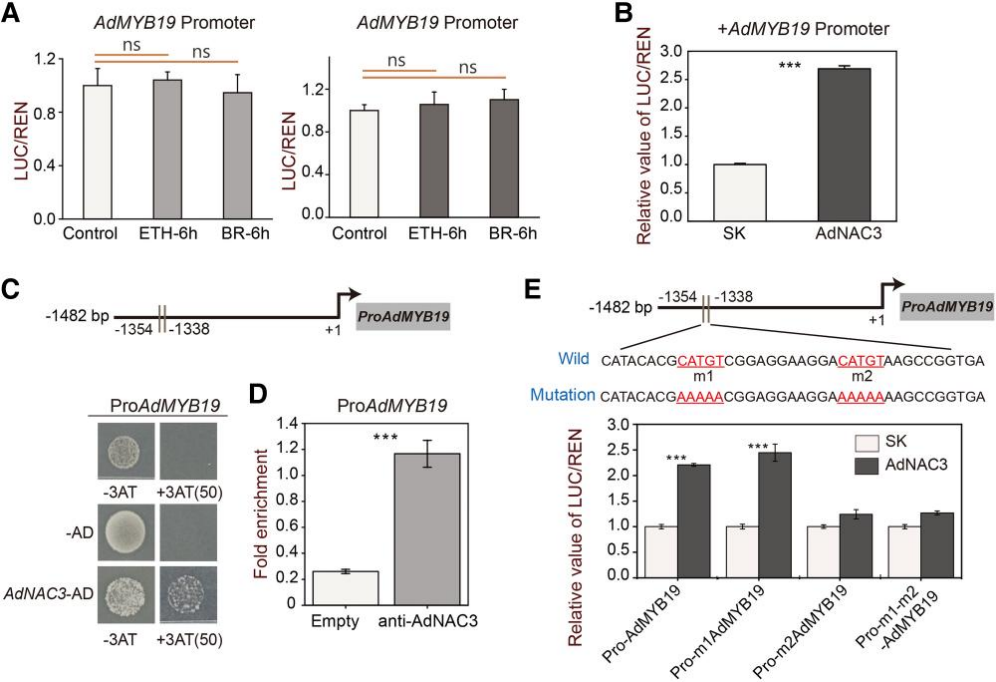
Regulatory effects of AdNAC3 on the promoter activities of *AdEXP3*. **A)** Verification in *N. benthamiana* system showing the effect of ETH and BR treatments (6 and 12 h) on the *AdMYB19* promoter activity. **B)** Dual-luciferase assay confirming that AdNAC3 can bind to the *AdMYB19* promoter, with the empty vector SK and promoter mixture used as a negative control (LUC/REN set to 1). **C)** Y1H assay indicating that AdNAC3 significantly activates the *AdMYB19* promoter in Sd media lacking Trp, Leu, and His, supplemented with 50 mm 3-AT. PGADT7 empty vector (AD) and *AdMYB19* promoter combination were used as negative controls. **D)** ChIP-qPCR experiment confirming the binding affinity of anti-AdNAC3 GFP antibody to specific regions of the *AdMYB19* promoter, with the empty vector (35S::GFP) used as a negative control. Error bars represent Se based on 3 biological replicates. **E)** Site-directed mutagenesis analysis validating the binding effect of AdNAC3 on the *AdMYB19* promoter. m1 represents the first mutated position, m2 represents the second mutated position, and -m1-m2 represents simultaneous mutation at both positions. The WT promoter sequence was used as a control. Error bars represent Se based on 3 biological replicates. Asterisks indicate significant differences determined by Student's *t*-test (***P < 0.001); n.s. indicates no significant difference.

Studies have shown that NAC can recognize the core CATGT motif-binding sites in DNA (Tran et al. 2004). Two CATGT-binding motifs were identified in the *AdMYB19* promoter region, suggesting that AdNAC3 may regulate the promoter sequence of *AdMYB19*. First, the dual-luciferase assay was employed to assess the results in the *N. benthamiana* transient expression system, which indicated that the *AdMYB19* promoter was not directly modulated by ETH and BR (Fig. 6A). Subsequently, the dual-luciferase assay analysis showed that AdNAC3 significantly activated the activity of the *AdMYB19* promoter by 2.69-fold (Fig. 6B). Y1H assays further demonstrated that AdNAC3 could bind to the *AdMYB19* promoter (Fig. 6C). The ChIP-qPCR results revealed that AdNAC3 significantly enriched the CATGT region of the *AdMYB19* promoter (Fig. 6D). Site-directed mutagenesis assays also indicated that AdNAC3 directly bound to the CATGT region of the *AdMYB19* promoter (Fig. 6E). Collectively, these results suggest that AdNAC3 can activate the transcription of *AdMYB19*.

**Figure 7.**
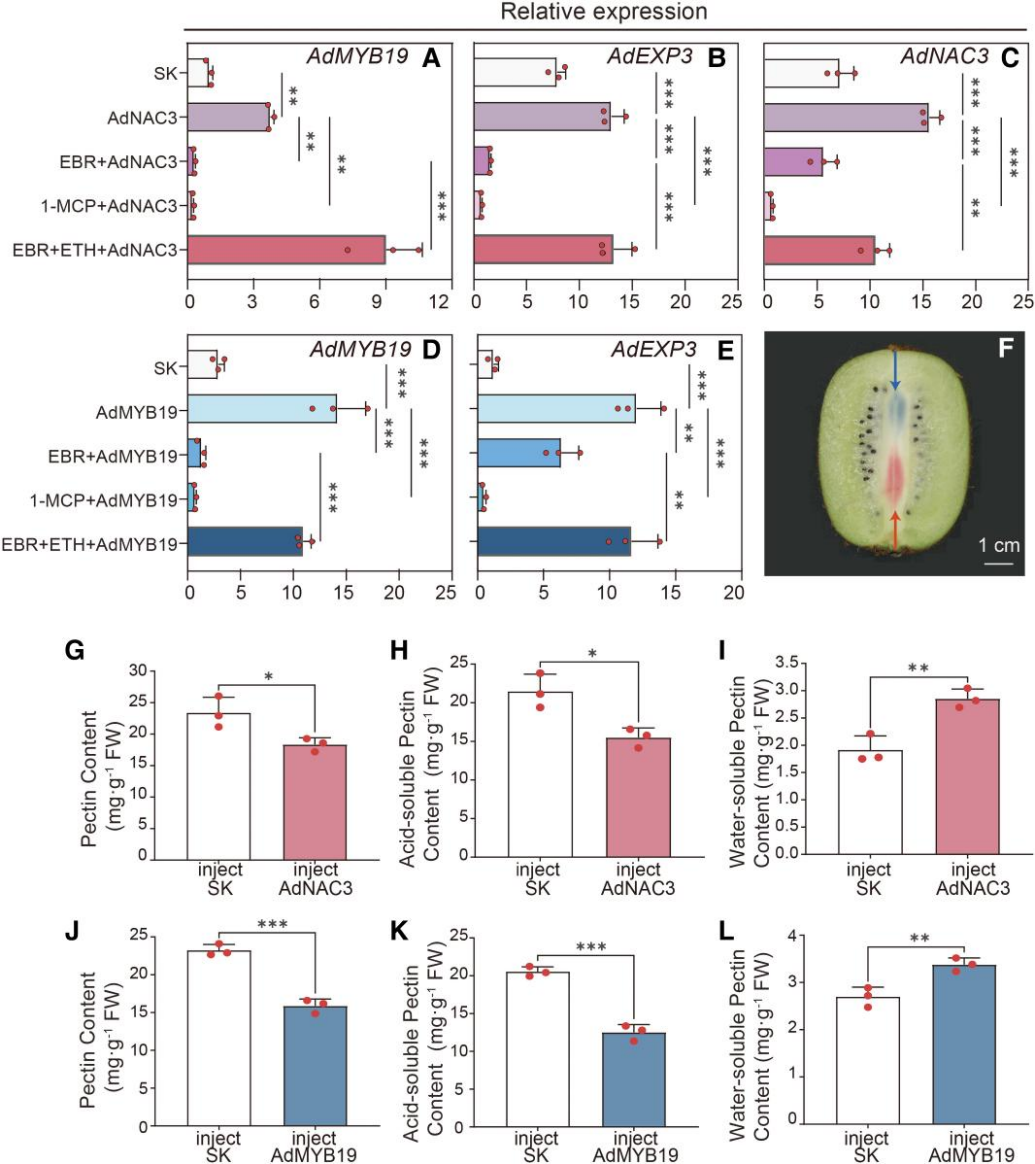
Functional validation of AdNAC3 and AdMYB19 in ‘Hayward’ kiwifruit by transient OE. Transient OE of *AdNAC3* and *AdMYB19* in kiwifruit. RT-qPCR analysis validated the transcriptional levels of **A)**  *AdMYB19*, **B)**  *AdEXP3*, and **C)**  *AdNAC3* in kiwifruit following transient OE of *AdNAC3*, as well as their transcriptional levels under EBR treatment, 1-MCP treatment, and the combined EBR and ETH treatment. RT-qPCR analysis validated the transcriptional levels of **D)**  *AdMYB19* and **E)**  *AdEXP3* in kiwifruit following transient OE of *AdMYB19* and compared their transcriptional levels under EBR treatment, 1-MCP treatment, and the combined EBR and ETH treatment. **F)** Schematic diagram for injection with differential color inks. The arrows show injection sites. The effects of transient OE of *AdNAC3* and *AdMYB19* on fruit softening-related parameters were measured, including changes in **G, J)** pectin, **H, K)** acid-soluble pectin, and **I, L)** WSP content. SK represents the empty vector (control). The error bars for the RT-qPCR data of *AdMYB19*, *AdEXP3*, and *AdNAC3* represent Se, based on 3 biological replicates. The error bars for the fruit softening indices, including pectin, acid-soluble pectin, and WSP contents, represent Se, based on 3 biological replicates. Asterisks indicate significant differences determined by Student's *t*-test (*P < 0.05, **P < 0.01, and ***P < 0.001). 1-MCP, 1-methylcyclopropene, an ETH receptor antagonist; EBR, brassinosteroid analog 2,4-epibrassinolide; ETH, ethylene; FW, fresh weight of fruit.

To explore the functions of AdNAC3 and AdMYB19 in kiwifruit, we performed transient OE and silencing experiments. The transient OE of *AdNAC3* significantly upregulated the expression of both *AdMYB19* and *AdNAC3*, with ETH treatment notably enhancing this effect and 1-MCP treatment significantly inhibiting it (Fig. 7, A to F). Furthermore, the transient OE of *AdNAC3* promoted pectin (Fig. 7G) and acid-soluble pectin (Fig. 7H) degradation and the accumulation of WSP (Fig. 7I). The transient OE of *AdMYB19* showed similar effects (Fig. 7, J to L). To further validate these results, we performed transient silencing of *AdNAC3* and *AdMYB19*, which yielded the opposite effect compared with the transient OE experiment (Supplementary Fig. S10). These findings further suggest that the expression of *AdNAC3* and *AdMYB19* is also upregulated by ETH, downregulated by BR, and involved in the fruit ripening process.

### Physical interaction between AdNAC3 and AdBBX32

Because both AdNAC3 and AdMYB19 can activate the expression of *AdEXP3*, we assessed their potential synergistic effect using dual-luciferase assays. The coexpression of AdNAC3 and AdMYB19 led to a synergistic activation of the *AdEXP3* promoter, resulting in a 5.5-fold increase. By contrast, the individual OE of AdNAC3 or AdMYB19 only resulted in 2.7-fold and 2.3-fold increases, respectively. No significant synergistic activation was observed with the individual expressions (Supplementary Fig. S11A). In addition, luciferase complementation imaging (LCI) and bimolecular fluorescence complementation (BIFC) assays further confirmed that there was no physical interaction between AdNAC3 and AdMYB19 (Supplementary Fig. S11, B and C).

**Figure 8.**
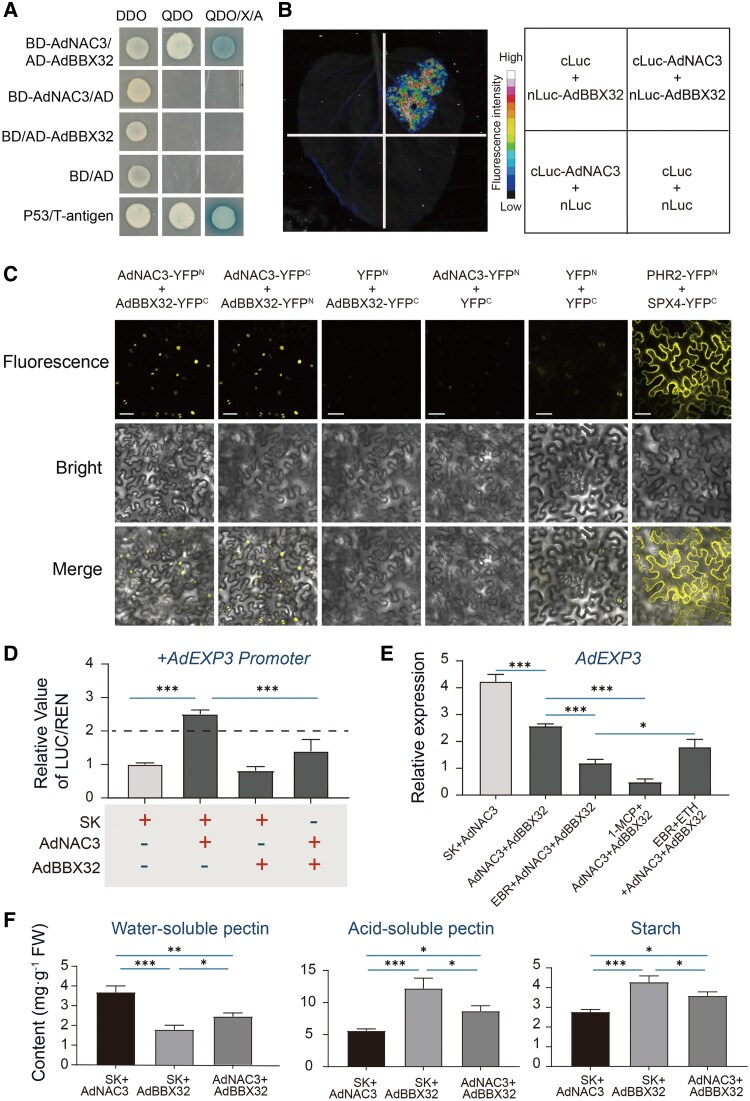
AdNAC3 physically interacted with AdBBX32. **A)** Y2H assay demonstrating the interaction between AdNAC3 and AdBBX32. The assay was performed on Sd media lacking Trp and Leu (DDO), Sd media lacking Trp, Leu, His, and Ade (QDO), and QDO/X/A media containing X-α-gal and aureobasidin A. Protein–protein interactions were detected on QDO and QDO/X/A plates. BD and AD vectors were used as negative controls, and P53/T-antigen was used as a positive control. **B)** Firefly LCI in *N. benthamiana* leaves. nLuc-AdBBX32 and cLuc-AdNAC3 fusion constructs were cotransformed into *N. benthamiana*, with nLuc-AdBBX32 + cLuc, cLuc-AdNAC3 + nLuc, and nLuc + cLuc used as negative controls. **C)** BiFC assay confirms the interaction between AdNAC3 and AdBBX32. The N-terminal and C-terminal YFP fragments (represented as YFPN and YFPC) were fused with the C-terminal and N-terminal of AdNAC3 and AdBBX32, respectively. YFPN and YFPC constructs with corresponding AdNAC3 and AdBBX32 vectors were used as negative controls. YFP fluorescence indicates protein-protein interaction. **D)** Dual-luciferase assay validating the regulatory effect of the AdNAC3-AdBBX32 interaction on the downstream target gene *AdEXP3*. **E)** RT-qPCR confirmed the effect of cotransient expression of *AdNAC3* and *AdBBX32* on the expression level of *AdEXP3* in kiwifruit. **F)** Measurement of the effect of cotransient OE of *AdNAC3* and *AdBBX32* on fruit softening indicators, including pectin, WSP, and acid-soluble pectin. The error bars for the relative value of LUC/REN represent Se based on 4 biological replicates. The error bars for the relative expression values and the values of WSP, acid-soluble pectin, and starch represent Se based on 3 biological replicates. SK represents the empty vector (control). Scale bar: 50 *μ*m. Asterisks indicate significant differences according to Student's *t*-test (*P < 0.05, **P < 0.01, and ***P < 0.001). 1-MCP, 1-methylcyclopropene, an ETH receptor antagonist; EBR, brassinosteroid analog 2,4-epibrassinolide; ETH, ethylene; FW, fresh weight of fruit.

To further investigate the regulatory network of AdNAC3 on kiwifruit ripening, we employed a yeast 2-hybrid (Y2H) library screening system to systematically identify putative interacting proteins of AdNAC3. Among the identified proteins, the BBX protein AdBBX32 showed physical interaction with AdNAC3 (Fig. 8A). Subcellular localization analysis revealed that AdBBX32 was also localized in the nucleus (Supplementary Fig. S12), and further colocalization experiments showed that AdNAC3 and AdBBX32 colocalized in the nucleus (Supplementary Fig. S13). RT-qPCR analysis indicated that the expression of *AdBBX32* was downregulated by BR but was not affected by ETH (Supplementary Fig. S14). LCI assays confirmed the physical interaction between AdNAC3 and AdBBX32 (Fig. 8B), and BIFC assays further validated this physical interaction (Fig. 8C). To assess the effect of the AdNAC3–AdBBX32 physical interaction on the regulation of downstream target genes, dual-luciferase assays showed that AdBBX32 weakened the regulatory effect of AdNAC3 on the *AdEXP3* promoter (Fig. 8D). These results indicate that AdNAC3 regulates the expression of *AdEXP3* through its physical interaction with the AdBBX32, which attenuates its regulatory effect on *AdEXP3*.

The transient OE of *AdBBX32* in kiwifruit further confirmed its role in the regulation of fruit ripening. The RT-qPCR analysis showed that the transcription levels of *AdBBX32* were significantly higher in the OE samples compared with the control (Supplementary Fig. S15A), and it notably downregulated the expression of *AdMYB19* and *AdEXP3* (Supplementary Fig. S15, B and C). The pectin content and acidic-soluble pectin levels in the *AdBBX32*-OE fruits were higher than those in the control, and the WSP content was lower (Supplementary Fig. S15, D to F). These results suggest that AdBBX32 negatively regulates kiwifruit ripening. The cotransient OE of *AdNAC3* and *AdBBX32* in fruits significantly suppressed the transcription of *AdEXP3* (Fig. 8E), reduced the accumulation of WSP, and delayed the degradation of acidic-soluble pectin and starch (Fig. 8F). These results support the AdBBX32, through its physical interaction with AdNAC3, alleviates the transcriptional activation of *AdEXP3* by AdNAC3, thereby delaying the fruit ripening process.

Given that the transcription level of *AdNAC3* was significantly upregulated by ETH treatment and downregulated by EBR treatment, we hypothesized that the promoter sequence of *AdNAC3* (Pro*AdNAC3*) may be coregulated by ETH and BR. A transient expression assay of Pro*AdNAC3*-GUS in kiwifruit demonstrated that the GUS enzyme activity of Pro*AdNAC3* was enhanced under ETH treatment but was significantly reduced under EBR treatment (Supplementary Fig. S16A). Furthermore, the expression level of *AdNAC3* was highly induced by ETH treatment but suppressed by EBR treatment (Supplementary Fig. S16B). These results further confirm that *AdNAC3* is positively regulated by ETH and negatively regulated by BR.

### OE of *AdNAC3* accelerated tomato fruit ripening

**Figure 9.**
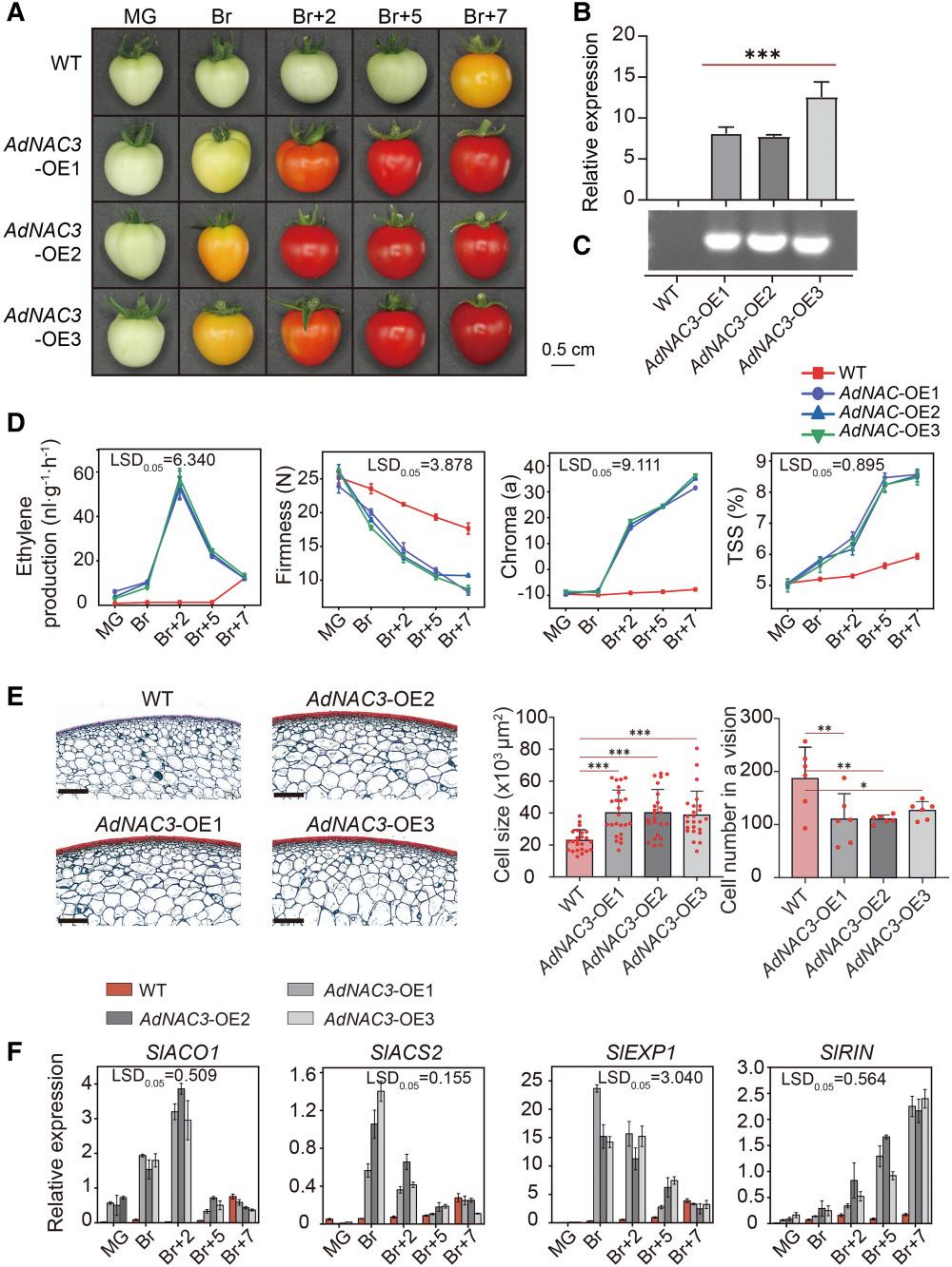
OE of *AdNAC3* promotes fruit ripening in tomato. **A)** Phenotypes of *AdNAC3* overexpressing fruits. Three independent *AdNAC3*-OE transgenic lines (OE-1, OE-2, and OE-3) and WT fruits at 5 different stages of ripening, from mature green to red: MG, mature green; Br, breaker; Br + 2, breaker plus 2 d; Br + 5, breaker plus 5 d; Br + 7, breaker plus 7 d. **B)** RT-qPCR analysis and **C)** RT-PCR analysis showing the transcriptional level of *AdNAC3* in fruits of WT and high-expressing *AdNAC3* lines. **D)** Changes in ETH production, fruit firmness, peel color, and TSS during fruit ripening in WT and *AdNAC3*-OE lines. Error bars represent means ± Se from 3 biological replicates. Lsd indicates significant differences as determined by the least significant difference test (*P* = 0.05). **E)** Microscopic observation of fruit at the Br stage in *AdNAC3*-OE lines and WT, along with statistics on cell size and cell number. Cell size (average value in 1 vision, *n* = 24) and cell number in 1 image (*n* = 6). The error bars for cell size and cell number represent Se based on 24 and 6 biological replicates, respectively. The cell shapes of WT, *AdNAC3*-OE1, *AdNAC3*-OE2, and *AdNAC3*-OE3 observed under a microscope all have a scale bar of 400 *µ*m. Asterisks indicate significant differences as determined by Student's *t*-test (*P < 0.05, **P < 0.01, and ***P < 0.001). **F)** Relative expression of ripening-related genes *SlACO1*, *SlACS2*, *SLEXP1*, and *SlRIN* in fruits of WT and *AdNAC3*-OE lines during the ripening stage. Gene expression levels were measured by RT-qPCR and normalized to the *SlActin*. Values are presented as means ± Se (*n* = 3). Lsd indicates significant differences as determined by the least significant difference test (*P* = 0.05). FW, fresh weight of fruit.

To explore the biological function of AdNAC3 in fruit ripening, we successfully generated 3 independent *AdNAC3*-OE lines, designated OE-1, OE-2, and OE-3 in ‘Micro-Tom’ tomatoes (Fig. 9A). The mRNA and DNA level analyses confirmed the successful expression of *AdNAC3* in the tomato lines (Fig. 9, B and C). Compared with wild-type (WT) tomato fruits, the *AdNAC3* transgenic fruits exhibited earlier ripening, as indicated by parameters such as fruit firmness, ETH production, color change, and TSS content (Fig. 9D). Studies have reported that EXPs are involved in the relaxation of cell walls by breaking hydrogen bonds, which promotes cell expansion and plant growth (Cosgrove 2005). Given that AdNAC3 positively regulated *AdEXP3*, we examined the microstructure of the fruit cells during ripening. Histological analysis revealed that compared with WT fruits, the cells of AdNAC3 overexpressing fruits were looser with a reduced cell number (Fig. 9E). In addition, we observed a significant upregulation of the expression of fruit ripening and ripening-related genes, including *SlACO1*, *SlACS2*, *SlEXP1*, and *SlRIN* in transgenic tomato fruits (Fig. 9F). These results indicate that AdNAC3 significantly promotes fruit ripening.

To elucidate the molecular mechanism by which AdNAC3 regulates fruit ripening, we performed RNA-seq analysis on the fruits of WT and *AdNAC3* transgenic tomato lines. PCA of the RNA-seq from WT and *AdNAC3*-OE fruits clearly separated the samples into 2 distinct groups, indicating good reproducibility of the data (Supplementary Fig. S17A). A total of 5,327 DEGs were identified, including 2,719 upregulated genes and 2,410 downregulated genes (Supplementary Fig. S17B). Further analysis of these DEGs revealed that most of the genes involved in cell wall modification were significantly upregulated in the *AdNAC3*-OE transgenic lines (Supplementary Fig. S17C). These results suggest that AdNAC3 regulates fruit ripening by upregulating the expression of genes involved in cell wall modification.

## Discussion

### BR delayed the ripening of kiwifruit by inhibiting the endogenous ETH biosynthesis and the degradation of cell wall components

Fruit ripening is the result of the crosstalk between various plant hormones, with BR and ETH playing critical roles in fruit ripening. In tomatoes, exogenous BR treatment promotes the expression of ETH biosynthesis genes (*LeACS2*, *LeACS4*, *LeACO1*, and *LeACO4*), thereby increasing ETH production and accelerating fruit ripening (Zhu et al. 2015). In bananas and mangoes, exogenous BR also promoted ETH production, accelerating fruit color change and ripening (Zaharah et al. 2012; Guo et al. 2019). In persimmons, exogenous BR treatment facilitated fruit ripening by regulating cell wall degradation and ETH biosynthesis (He et al. 2018). However, in apples and pears, exogenous BR inhibited ETH production and delayed fruit ripening (Ji et al. 2021). Our study also shows that exogenous EBR treatment delayed the ETH production and slowed down the reduction in fruit firmness, thereby delaying the ripening process of kiwifruit, whereas ETH treatment accelerated the ETH production and promoted fruit ripening (Fig. 1). The combined treatment of ETH and BR further indicates that BR antagonizes ETH in the regulation of kiwifruit ripening. This result is consistent with previous studies, which showed that exogenous BR treatment delayed the decrease in fruit firmness and delayed kiwifruit ripening and ETH treatment accelerated ripening (Zhang et al. 2018; Wang et al. 2020). These findings suggest that exogenous BR treatment inhibits ETH production in kiwifruit, thereby delaying ripening and further supporting the antagonistic effects of ETH and BR in the regulation of kiwifruit ripening.

Studies have shown that the activities of Cel and PG enzymes cause cell wall degradation in kiwifruit, leading to fruit softening (Xiong et al. 2023). Our study demonstrates that ETH treatment significantly promoted the activity of cell wall-degrading Cel and PG enzymes, resulting in a decrease in fruit firmness, and 1-MCP and EBR treatments significantly reduced the activity of these enzymes (Fig. 1). This conclusion is further validated in peaches, pears, and tomatoes (Shi et al. 2022). During postharvest storage, starch content in kiwifruit not only affects fruit ripening but also influences flavor. As storage time increases, starch is gradually degraded and converted into soluble sugars (Harker et al. 2009). The rapid increase in amylase activity leading to starch hydrolysis is an essential factor in kiwifruit storage (Yan et al. 2023). Our results show that ETH treatment accelerated pectin and starch degradation during postharvest storage of kiwifruit, promoting the accumulation of TSS, whereas EBR treatment delays this process (Fig. 1). The phenotype resulting from the combined ETH and BR treatment lies between those of ETH and BR treatments alone. In addition, through WGCNA, we identified potential cell wall-related genes, namely, *AdEXP3*, *AdPME1*, and *Adβ-Gal5*, that may be involved in kiwifruit ripening (Fig. 2). The expression of these genes was significantly upregulated by ETH and downregulated by BR (Fig. 4), showing a gradual increase during fruit ripening, which was negatively associated with changes in fruit firmness. Conversely, silencing *SlPG2a* and *SlEXP1* in tomatoes significantly increases fruit firmness (Powell et al. 2003; Uluisik et al. 2016; Yang et al. 2017). These findings further suggest that ETH and BR regulate kiwifruit ripening by modulating the expression of cell wall-related genes and the degradation of cell wall components.

### AdNAC3–AdMYB19 transcriptional regulatory cascade accelerated kiwifruit ripening by regulating *AdEXP3*

NAC plays a crucial role in regulating fruit ripening and senescence by modulating the expression of ripening-related genes (Wei, Liu, et al. 2023; Zhang et al. 2024). In peaches, PpNAC1 and PpNAC5 activate the transcription of cell wall degradation genes, including *PpCEL3*, *PpPMEI3*, *PpXET23*, *PpXET33*, *PpPG1*, and *PpPG1*2, thereby regulating fruit ripening (Zhang et al. 2024). In apples, MdNAC72 participates in fruit ripening by regulating the expression of *MdPG1* (Wei, Yang, et al. 2023). In kiwifruit, the zinc finger TF AdZAT5 participates in fruit ripening regulation by activating the expression of pectin degradation genes, namely, *AdPL5* and *Adβ-Gal5* (Zhang et al. 2022). We also identified an NAC TF, AdNAC3, which directly activated the expression of *AdEXP3* (Fig. 3). In transient OE experiments, *AdNAC3* significantly upregulated *AdEXP3* expression and promoted fruit ripening (Fig. 7), confirming its regulatory role in kiwifruit. The phenotype of transient silencing *AdNAC3* further validated the transient OE results (Supplementary Fig. 10). In addition, the stable OE of *AdNAC3* in tomatoes accelerated fruit ripening and promoted cell expansion, further demonstrating that AdNAC3 positively regulates fruit ripening (Fig. 9). These findings are consistent with previous research in strawberries, where the NAC TF FvRIF regulates fruit ripening through the modulation of *FvEXP3*, *FvPG2*, and *FvPL2* (Li, Martín-Pizarro, et al. 2023). Collectively, these studies suggest that AdNAC3 directly regulates the ripening-related target gene *AdEXP3* and is involved in the softening of kiwifruit. This work lays the foundation for future studies on the molecular regulatory network controlling kiwifruit ripening. However, the transcriptional regulatory mechanisms and molecular functions of *AdPME1* and *Adβ-Gal5* still require further investigation.

Fruit ripening is a complex and precisely regulated network, where various TFs play crucial roles, and the transcriptional regulatory mechanisms involved have been extensively elucidated (Liu et al. 2022). However, transcriptional cascade regulation in fruit ripening has been less reported. Recent studies have shown that in bananas, MaNAC083 regulates the expression of ETH biosynthesis-related genes, including *MaACS1*, *MaACO1*, *MaACO4*, *MaACO5*, and *MaACO8*. Furthermore, MaMADS1 has been found to regulate the expression of *MaNAC083*, forming a MaMADS1–MaNAC083 transcriptional cascade that modulates banana ripening (Wei, Yang, et al. 2023). In peaches, a PpEIL2/3–PpNAC1–PpWRKY14 transcriptional cascade has also been identified, which regulates fruit ripening (Liu et al. 2024). However, the transcriptional cascade regulation upstream of cell wall degradation-related genes during kiwifruit ripening remains unclear. In this study, we found that AdNAC3 directly regulates the expression of *AdMYB19* (Fig. 6) and AdMYB19 subsequently activates the expression of *AdEXP3* (Fig. 5). Therefore, a transcriptional cascade involving AdNAC3–AdMYB19 regulates the cell wall gene *AdEXP3*, thereby participating in kiwifruit ripening. This mechanism is similar with that observed in tomatoes, where SlWOX13 regulates the expression of ripening-related TFs such as *SlRIN*, *SlNOR*, and *SlNAC4*, contributing to fruit ripening. In fruit ripening, the NAC family primarily regulates the expression of ripening-related target genes through its members. Our findings suggest that AdNAC3 not only directly regulates *AdEXP3* but also reveals a mechanism in which the AdNAC3–AdMYB19 transcriptional cascade is involved in kiwifruit ripening. Although this study provides essential insights into the role of AdNAC3 in the ripening process of kiwifruit, several issues warrant further investigation. First, the research primarily focuses on *AdEXP3*, and the regulation of other ripening-related genes requires additional exploration. Moreover, the molecular mechanisms upstream of AdNAC3 regulation have yet to be elucidated, necessitating more comprehensive studies employing molecular biology techniques in the future.

### AdNAC3 and AdBBX32 involved in kiwifruit ripening via protein–protein interaction

TFs activate or inhibit the expression of target genes through interactions, and their ability to bind to target gene promoters depends on their concentration or molecular partners (Cao et al. 2024). Studies have found that NAC TFs can form heterodimers with other proteins. For example, in apples, MdNAC7 physically interacts with MdWRKY31 to form a complex that prevents MdWRKY31 from binding to the *MdXTH2* promoter, thereby slowing down fruit ripening (Wang et al. 2024). In bananas, MaNAC1 and MaNAC2 physically interact with the ETH signaling factor MaEIL5, participating in the regulation of fruit ripening (Shan et al. 2012). In papayas, CpMADS4 physically interacts with CpNAC3, enhancing the regulation of ETH signaling genes *CpERF9* and *CpEIL5*, thus promoting fruit ripening (Fu, Chen, et al. 2021). In tomatoes, SlNAC4 physically interacts with SlRIN and SlNOR, regulating fruit ripening (Zhu et al. 2014). However, the role of BBX proteins in regulating changes in fruit texture has been less explored. This study found that OE of *AdBBX32* slowed down pectin degradation, indicating that AdBBX32 acts as a negative regulator of fruit ripening (Supplementary Fig. S15). This finding is consistent with the recent discovery that MdBBX25 negatively regulates fruit ripening (Zhang et al. 2024). Furthermore, our research shows that AdNAC3 physically interacts with AdBBX32, and this interaction attenuates the regulatory effect of AdNAC3 on *AdEXP3* (Fig. 8). We hypothesize that the physical interaction between AdBBX32 and AdNAC3 modulates the activity of AdNAC3 through multiple mechanisms, including competing for DNA-binding sites, promoting AdNAC3 degradation, and interfering with its interactions with auxiliary TFs, thereby reducing its transcriptional activation capacity. In future studies, we will experimentally validate the impact of AdBBX32 on the binding of AdNAC3 to target promoters using a variety of experimental approaches, assess its regulatory role through protein stability experiments, and explore the specific mechanisms of AdBBX32 in the ETH and BR signaling networks via gene knockout or OE approaches. This comprehensive approach aims to elucidate the regulatory networks governing fruit maturation and softening.

NAC has been widely reported to play a role in regulating fruit ripening (Liu et al. 2022). Promoter activity analysis has revealed the gene's response to hormones. In kiwifruit, the expression of *AcGH3.1* was significantly upregulated under ETH treatment, and promoter activity assays indicated a higher GUS enzyme activity under ETH treatment (Gan, Yuan, Shan, Wan, Chen, Zhu, et al. 2021). Our study found that the expression level of *AdNAC3* was significantly upregulated under ETH treatment, and it was notably downregulated under EBR treatment (Fig. 4). GUS enzyme activity assays of the *AdNAC3* promoter sequence showed that ETH treatment increased GUS activity, whereas EBR treatment inhibited GUS activity (Supplementary Fig. S16). This further suggests that the expression of *AdNAC3* was coregulated by both ETH and BR. This finding is similar with the role of MaNAC083 as a negative ETH response factor, where ETH treatment decreased the activity of the *MaNAC083* promoter in bananas (Wei, Yang, et al. 2023). These results suggest that AdNAC3 may be involved in the interaction between ETH and BR.

## Conclusions

**Figure 10.**
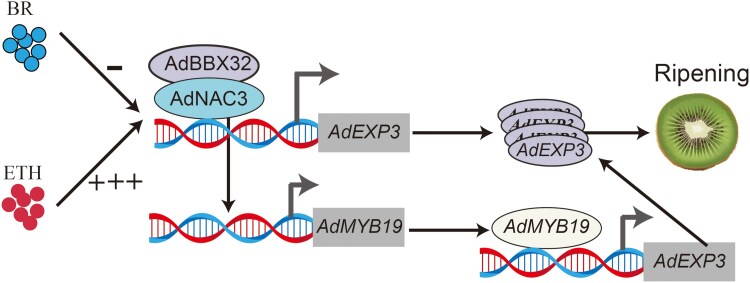
The model of AdNAC3 and AdMYB19 regulates fruit ripening. ETH promoted the expression of the *AdNAC3* in kiwifruit, whereas BR inhibits *AdNAC3* expression. AdNAC3 affects fruit softening through multiple mechanisms: firstly, AdNAC3 and AdMYB19 directly regulate the expression of the *AdEXP3*, promoting *AdEXP3* transcription and thereby promoting fruit softening. Additionally, AdNAC3 regulates *AdMYB19*, which in turn modulates *AdEXP3* expression to further promote fruit softening. Furthermore, AdNAC3 interacts with AdBBX32 to further regulate kiwifruit softening. In the model, “−” represents inhibition and “+” represents promotion. BR, brassinosteroid; ETH, ethylene.

We determined that the expression of *AdNAC3* and *AdMYB19* was upregulated by ETH and downregulated by BR. Furthermore, we found that AdMYB19 and AdNAC3 cooperated to regulate the expression of *AdEXP3*, thereby participating in the fruit ripening process. AdNAC3 regulated the transcriptional cascade of *AdMYB19*, which, in turn, controlled *AdEXP3*, and AdNAC3 physically interacted with AdBBX32 to participate in the regulation of fruit ripening (Fig. 10). These findings enhance our understanding of the regulatory mechanisms underlying kiwifruit softening during ripening and provide a theoretical foundation for developing storage-resistant varieties and studying the hormonal crosstalk regulating fruit softening.

## Materials and methods

### Plant materials and treatments

Mature fruits of ‘Hayward’ kiwifruit (*Actinidia deliciosa* cv. ‘Hayward’) with a TSS content > 6.0% were harvested from a commercial orchard in Xianyang, Shaanxi Province, China. The fruits were free from mechanical injury and were uniform in size. Selected fruits were subjected to treatments in 2021, including ETH (100 *μ*L/L), 1-MCP (1 *μ*L/L), and a control group (air), with a 24-h exposure period. In addition, for the BR treatment, 10 *μ*m EBR was applied with a 0.1% Tween-20 (*v*/*v*) solution every 0, 6, 12, and 18 h. The control group was treated with the same solution of 0.1% Tween-20 (*v*/*v*) sterile water. After treatment, the fruits were allowed to air dry. In 2022, to further investigate the role of BR and ETH in kiwifruit ripening, a combined treatment was conducted. In the EBR + ETH (EBR + ETH) treatment, fruits were first treated with 10 *μ*m EBR, followed by ETH (100 *μ*L/L). In the ETH + EBR (ETH + EBR) treatment, fruits were first treated with ETH (100 *μ*L/L), followed by 10 *μ*m EBR. After treatment, the fruits were stored at room temperature (20 °C), with 260 fruits per treatment group. For each sampling point, 3 biological replicates were taken, for a total of 12 fruits. Fruit samples were quickly frozen in liquid nitrogen and stored at −80 °C for further analysis.

### Fruit physiological properties

Fruit firmness was measured using a texture analyzer (GS-15, Germany). Briefly, 2 peel segments were removed at a 90° angle from the equatorial regions of fruits, and the average firmness value was recorded. A total of 12 fruits were measured at each time point.

The TSS contents of the fruits were measured using an ATAGO refractometer (PAL-BX/ACID 8, Tokyo, Japan). Briefly, the juice was extracted by squeezing 3 drops from both ends of the fruit's flesh, which were then mixed thoroughly for measurement. A total of 12 biological replicates were analyzed at each sampling point.

The ETH production from the fruit was measured using a gas chromatograph (GC-14A, Tokyo, Japan). Briefly, kiwifruits were placed in a sealed desiccator and sealed for 1 h. Afterward, 1 mL of gas was extracted, with 3 biological replicates per treatment. ETH content was then determined using the gas chromatograph.

The determination of starch content, cell wall-related substances, and cell wall enzyme activities was based on previously established methods with minor modifications (Zhang et al. 2018; Xiong et al. 2023).

### RNA-seq and WGCNA analysis

RNA-seq and bioinformatics analyses were performed by Biomarker Technologies (Beijing, China). RNA-seq was used to sequence the transcriptomes of the kiwifruit samples treated with ETH, 1-MCP, and EBR at the same time point, aiming to observe gene expression changes under different treatments and at different time points during the ripening process. Three biological replicates were used for each sample at each time point. Sequencing libraries were constructed and sequenced using the Illumina NovaSeq 6000 platform. The reference genome was from the kiwifruit ‘Red5’ (Pilkington et al. 2018; Yue et al. 2020). DEGs were identified with |log2 FC| > 1 and a significance *P* < 0.05.

WGCNA analysis was employed to identify key TFs and structural genes involved in fruit ripening, which are simultaneously induced by ETH and BR. The specific procedures for WGCNA followed previous studies (Li, Martín-Pizarro, et al. 2023). Briefly, WGCNA was conducted based on the fragments per kilobase of transcript per million mapped reads (FPKM) values of all DEGs from the transcriptome data, along with the combined physiological phenotypes. The analysis was performed using R (v1.69), and the regulatory network was visualized using Cytoscape (v3.8.2).

### Dual-luciferase and site-directed mutagenesis assays

The dual-luciferase assay was used to investigate the regulatory effects of different TFs on the promoters of cell wall degradation-related genes. Briefly, the full-length coding sequences (CDS) of 19 TFs and the promoters of 3 cell wall degradation-related genes (*AdEXP3*, *AdPME1*, and *Adβ-Gal5*) were individually cloned into the pGreen II 0029 62-SK and pGreen II 0800-Luc vectors. The recombinant vectors were then transformed into *Agrobacterium tumefaciens* strain GV3101 (pSoup), and the bacterial suspension was prepared in an infiltration buffer (ddH_2_O, 10 mm MES, 10 mm MgCl_2_, 150 *μ*m acetosyringone, pH 5.6) to OD600 of 0.75. The TFs and promoters were mixed in a 10:1 (*v*/*v*) ratio and injected into the leaves of *N. benthamiana*. After 3 d, a dual-luciferase reporter gene assay kit (Yeasen Biotech, China) was used to measure chemiluminescence, and values were read using an Infinite M200Pro microplate reader (Tecan, Switzerland). The LUC/REN ratio was calculated and recorded. Four biological replicates were used to evaluate the experimental results. For the analysis of *AdPME1*, *AdEXP3*, and *Adβ-Gal5*, *Agrobacterium* cells were suspended in the same infiltration buffer and cultured to OD600 of 0.2. After 48 h of injection, the *N. benthamiana* leaves were sprayed with either water as a control or with 1,000-mg/kg ethephon and 10 *μ*m EBR. The primers used for vector construction are listed in Supplementary Table S2.

Site-directed mutagenesis assays were performed to further confirm the binding sites of TFs on target gene promoters. First, primers containing mutation sites in the *AdEXP3* and *AdMYB19* promoter sequences were designed, and the mutated promoter sequences were cloned into the pGreen II 0800-Luc vector. Then, using the same procedure as the dual-luciferase assay, the effects of point mutations on target gene promoter activity were evaluated based on the LUC/REN ratio, thereby confirming the functional role of the mutation sites in gene regulation.

### Y1H assay and ChIP-qPCR analysis

Y1H assays were conducted to validate the binding of AdMYB19 and AdNAC3 to the *AdEXP3* promoter, as well as the effect of AdNAC3 binding to the *AdMYB19* promoter. Briefly, the CDS of *AdMYB19* was cloned into the pGADT7 vector, and the promoter sequences of *AdEXP3* and *AdMYB19* were constructed into the pHIS2 vector. These constructs were then transformed into the yeast strain Y187. The detailed protocol for Y1H assays followed previous studies (Wei, Liu, et al. 2023).

The ChIP-qPCR was performed following the methodology described in previous studies (Zhu et al. 2023). Briefly, chromatin was extracted from kiwifruit samples that had been sonicated for random fragmentation. Immunoprecipitation was conducted using anti-GFP antibodies specific to AdMYB19 and AdNAC3, and the chromatin complexes were analyzed by RT-qPCR with 3 biological replicates. An empty vector was used as a negative control. The primers for Y1H and ChIP-qPCR are listed in Supplementary Table S2.

### Y2H assay

To identify proteins interacting with AdNAC3, a Y2H system (Matchmaker Gold Yeast Two-Hybrid System, Clontech, USA) was used according to the manufacturer's instructions. The CDS of *AdNAC3* was cloned into the pGBKT7 vector to construct the bait vector pGBKT7–AdNAC3, which was then transformed into the yeast strain AH109. After confirming the absence of self-activation and toxicity, further screening was conducted. The cDNA library constructed from kiwifruit at the mature stage was used for the Y2H assay (Make Your Own “Mate & Plate” Library System, Clontech, USA). The library plasmids were mated with the yeast strain carrying the bait vector and plated onto selective media lacking Trp, Leu, His, and Ade (SD/-Trp–Leu–His–Ade, Clontech, USA). The plates were incubated at 28 °C for 3 to 5 d, and positive clones were selected. Plasmids from positive clones were extracted and sequenced to identify candidate proteins interacting with AdNAC3. To validate the interaction, the CDS of the candidate TF *AdBBX32* was cloned into the pGADT7 vector and cotransformed with pGBKT7–AdNAC3 into the AH109 strain, following the same procedure as described above. The primers for Y2H are listed in Supplementary Table S2.

### Subcellular localization assay and BIFC assay

The full-length CDS of *AdMYB19*, *AdNAC3*, and *AdBBX32* with the stop codons removed were cloned into the pCAMBIA2300-EGFP vector to construct fusion expression vectors. These fusion vectors were then transformed into *A. tumefaciens* strain GV3101, which was used to inoculate *N. benthamiana* leaves expressing mCherry for nuclear localization. After 48 h, fluorescence was observed using an inverted laser scanning microscope (Lecia TCS SP8, Germany) equipped with a 40×/1.4 water immersion objective. GFP was excited with a 488-nm solid-state laser, and fluorescence was detected within the 498 to 540 nm range, with an intensity of 4.9% and gain set to 800. For mCherry, excitation was performed using a 552-nm solid-state laser, and fluorescence was detected between 590 and 640 nm, with intensity at 9.1% and gain at 800. Pinholes were set to 1 airy unit for all wavelengths. Image processing was carried out using Leica LAS X software (Version 3.7.2) and ImageJ 1.8.0.

The BIFC assay was used to verify the physical interactions between AdNAC3 and AdBBX32. Briefly, the full-length *AdNAC3* and *AdBBX32* were cloned into the C-terminal and N-terminal fragments of the YFP vector, respectively. YFP signals were observed using a laser scanning confocal microscope according to the subcellular localization protocol. The primers for the subcellular localization assay and BIFC assay are listed in Supplementary Table S2.

### Firefly LCI assay

The vectors carrying nLUC-AdBBX32 and AdNAC3-cLUC were transformed into *A. tumefaciens* strain GV3101. The bacterial suspension was adjusted to OD600 of 1 and mixed in a 1:1 ratio before being injected into the leaves of *N. benthamiana*. Two days post-injection, 0.2 mm luciferin was applied to the injection sites, and after 30 min of dark incubation, Luc fluorescence signals were detected using a live plant imaging system (Lumazone Pylon 2048B, USA). The primers for LCI are listed in Supplementary Table S2.

### Transient OE and silencing in kiwifruit fruits

The transient OE and silencing methods in the core tissue of ‘Hayward’ kiwifruit were used to investigate the regulatory roles and functions of AdNAC3 and AdMYB19 (Zhang et al. 2018). Briefly, the overexpressed and silenced *AdNAC3* and *AdMYB19* were inserted into the pGreen II 0029 62-SK and pTRV2 vectors, respectively. Empty vectors and overexpressed and silenced *AdNAC3* and *AdMYB19* were injected into the core tissue of ‘Hayward’ kiwifruit using the same infiltration solution as the dual-luciferase assay. One day after injection, fruits were treated with 10 *μ*m EBR, 100-*μ*L/L ETH, or 1-*μ*L/L 1-MCP. The injected fruits were then stored at 20 °C for 3 d, and samples were collected in liquid nitrogen. Each fruit was considered a biological replicate, and samples were stored at −80 °C for RNA extraction.

The GUS enzyme activity assay of the *AdNAC3* promoter was conducted to further analyze the hormone responsiveness of *AdNAC3*. In brief, the *AdNAC3* promoter sequence was cloned into the PC0390-GUS vector. Subsequently, the recombinant vector was infiltrated into the core tissues of kiwifruit using the same method employed for transient OE, followed by treatment with ETH and EBR. GUS histochemical staining was performed using the GUSblue kit (Waryong, China), and GUS activity was quantitatively analyzed utilizing the Bradford protein assay kit and the GUS gene quantification detection kit (Coolaber, China). Three biological replicates were used to evaluate the experimental results.

### Tomato genetic transformation

To further investigate the function of AdNAC3, OE of *AdNAC3* was performed in tomatoes (Micro-Tom). Briefly, the CDS of *AdNAC3* was cloned into the pCAMBIA2300 vector and transformed into *A. tumefaciens* strain EHA105. The recombinant plasmid was then introduced in tomato plants following previously described protocols (Jiang et al. 2022). Transgenic tomato plants were selected based on the detection of *AdNAC3* at both mRNA and DNA levels. Three independent positive transgenic lines (T1) were selected for subsequent analysis.

Fruits of AdNAC3 transgenic tomatoes at the breaker stage were used for cytological analysis. Briefly, after harvesting the tomato fruits, longitudinal sections were carefully made with a scalpel, and the samples were fixed in FAA fixative (5% formaldehyde, 5% acetic acid, and 50% ethanol). The fixed samples were then dehydrated using a series of alcohol gradients, followed by clearing in xylene and embedding in paraffin. The paraffin-embedded sections were stained with Safranin O-Fast Green (G1031, Servicebio, Wuhan, China) to observe the cellular structure. The stained sections were imaged using a Leica MZ10F microscope (Germany). The images were analyzed for cell area and number using ImageJ software (Schneider et al. 2012). Images of the same size were imported into ImageJ, and the cell boundaries were identified using the segmentation tool. The area of each cell was calculated, and the number of cells per unit area was quantified.

### RT-qPCR analysis

RT-qPCR was performed as described in previous studies (Yang, Chen, et al. 2022; Yang, Shan, et al. 2022; Yang et al. 2024). Total RNA was extracted from kiwifruit using a modified CTAB method. cDNA was synthesized using the TaKaRa reverse transcription kit (Code No. RR047A, Takara, Japan). Specific primers for the target genes were designed based on the kiwifruit ‘Red5’ genome, and primer specificity was confirmed by agarose gel electrophoresis. RT-qPCR was conducted using LightCycler 480 (Roche, Germany) and LightCycler 480 SYBR Green I Master (Roche, Germany). Gene expression levels were calculated using the 2^−ΔCt^ method, with each experiment performed in triplicate biological repeats. The primers used for RT-qPCR are listed in Supplementary Table S2.

### Statistical analysis

Data were compared using ANOVA and the least significant difference at *P* = 0.05 using Microsoft Excel 2023. Subsequently, OriginPro 2018 was utilized for data visualization, and Adobe Illustrator CC 2021 was employed for image postprocessing.

### Accession numbers

Sequence data from this article can be found in the GenBank/EMBL data libraries under the following accession numbers: AdNAC3 (PV094659), AdMYB19 (PV094660), AdEXP3 (PV094661), AdPME1 (PV094662), AdBBX32 (PV094663), and Adβ-Gal5 (MG857556).

## Supplementary Material

kiaf084_Supplementary_Data

## Data Availability

The transcriptome data in this article are publicly available at NCBI: PRJNA1222169.

## Supplementary data

The following materials are available in the online version of this article.

**Supplementary Figure S1.** The impact of exogenous EBR treatment on endogenous BR levels in fruit.

**Supplementary Figure S2.** GO and KEGG enrichment analyses were performed on the RNA-seq under different treatments.

**Supplementary Figure S3.** Clustering and correlation analyses based on transcriptome data.

**Supplementary Figure S4.** Promoter activity analysis of functional genes.

**Supplementary Figure S5.** Expression levels of other TF genes during the ripening process of “Hayward” fruits following EBR treatment.

**Supplementary Figure S6.** Expression levels of other TF genes during the ripening process of ‘Hayward’ fruits following ETH treatment.

**Supplementary Figure S7.** Expression levels of *AdEXP3*, *Adβ-Gal5*, *AdPME1*, *AdMYB19*, and *AdNAC3* during the ripening process of ‘Hayward’ fruits following individual treatments of ETH and EBR, as well as the combined treatment of ETH and EBR (EBR + ETH and ETH + EBR).

**Supplementary Figure S8.** Subcellular localization analysis of AdMYB19 and AdNAC3.

**Supplementary Figure S9.** Verification of the function of AdEXP3 in ‘Hayward’ kiwifruit.

**Supplementary Figure S10.** Transient silencing validation of AdNAC3 and AdMYB19 functions in ‘Hayward’ fruits.

**Supplementary Figure S11.** AdNAC3 and AdMYB19 do not physically interact at the protein level.

**Supplementary Figure S12.** Subcellular localization analysis of TF AdBBX32.

**Supplementary Figure S13.** Subcellular colocalization analysis of AdNAC3 and AdBBX32.

**Supplementary Figure S14.** Expression levels of *AdBBX32* under ETH and EBR treatment conditions.

**Supplementary Figure S15.** Transient OE validation of AdBBX32 function in ‘Hayward’ kiwifruit.

**Supplementary Figure S16.** Transient OE of the *AdNAC3* promoter in kiwifruit to validate its promoter activity.

**Supplementary Figure S17.** RNA-seq analysis on the 7-d break stage (Br + 7) revealed the molecular basis of fruit ripening in the stable OE ‘Micro-Tom’ transgenic lines of *AdNAC3*.

**Supplementary Table S1.** Summary of RNA-seq data.

**Supplementary Table S2.** Primers used in this study.

## Dive Curated Terms

The following phenotypic, genotypic, and functional terms are of significance to the work described in this paper:

fruit AmiGo: PO:0009001

BBX Gramene: B-box protein
BBX Araport: B-box protein

WGCNA Gramene: weighted gene coexpression analysis
WGCNA Araport: weighted gene coexpression analysis
