# Supplementary material for: The transcription factors AdNAC3 and AdMYB19 regulate kiwifruit ripening through brassinosteroid and ethylene signaling networks
Source: Plant Physiol. 2025 Feb 20;197(3):kiaf084. doi: 10.1093/plphys/kiaf084 (PMC11896978; doi:10.1093/plphys/kiaf084)
Supplement: kiaf084_Supplementary_Data [file kiaf084_supplementary_data.zip › Supplementary Data.pdf]

Supplementary Data

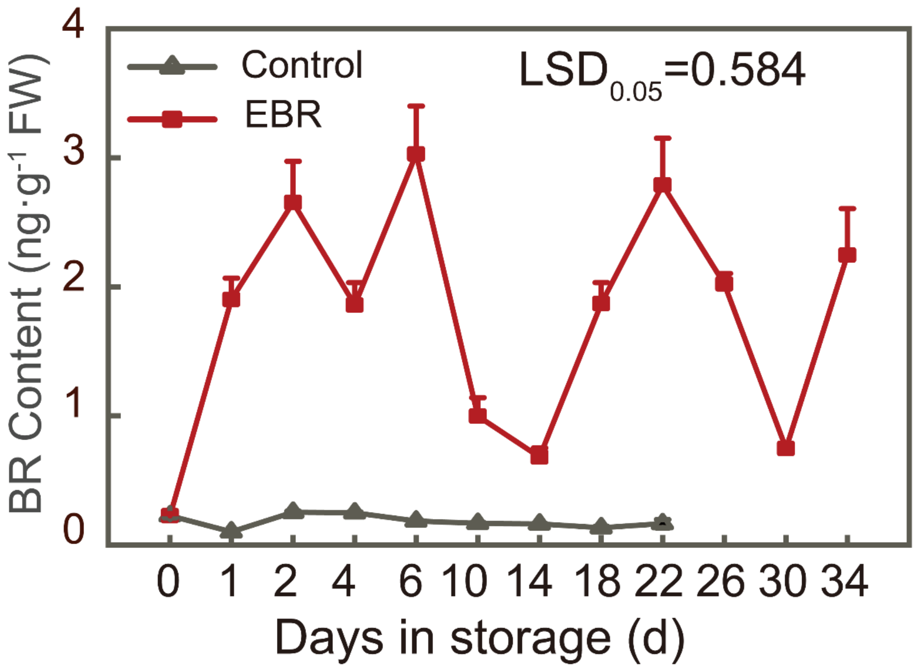

Supplementary Figure S1. The impact of exogenous EBR treatment on endogenous BR levels in fruit. Changes in endogenous BR content in 'Hayward' fruits subjected to EBR treatment at various storage times. EBR, brassinosteroid analog 2,4-epibrassinolide. FW, fresh weight of the fruit. Error bars represent SE based on 3 biological replicates. LSD indicates significant differences by least significant difference method (P = 0.05).

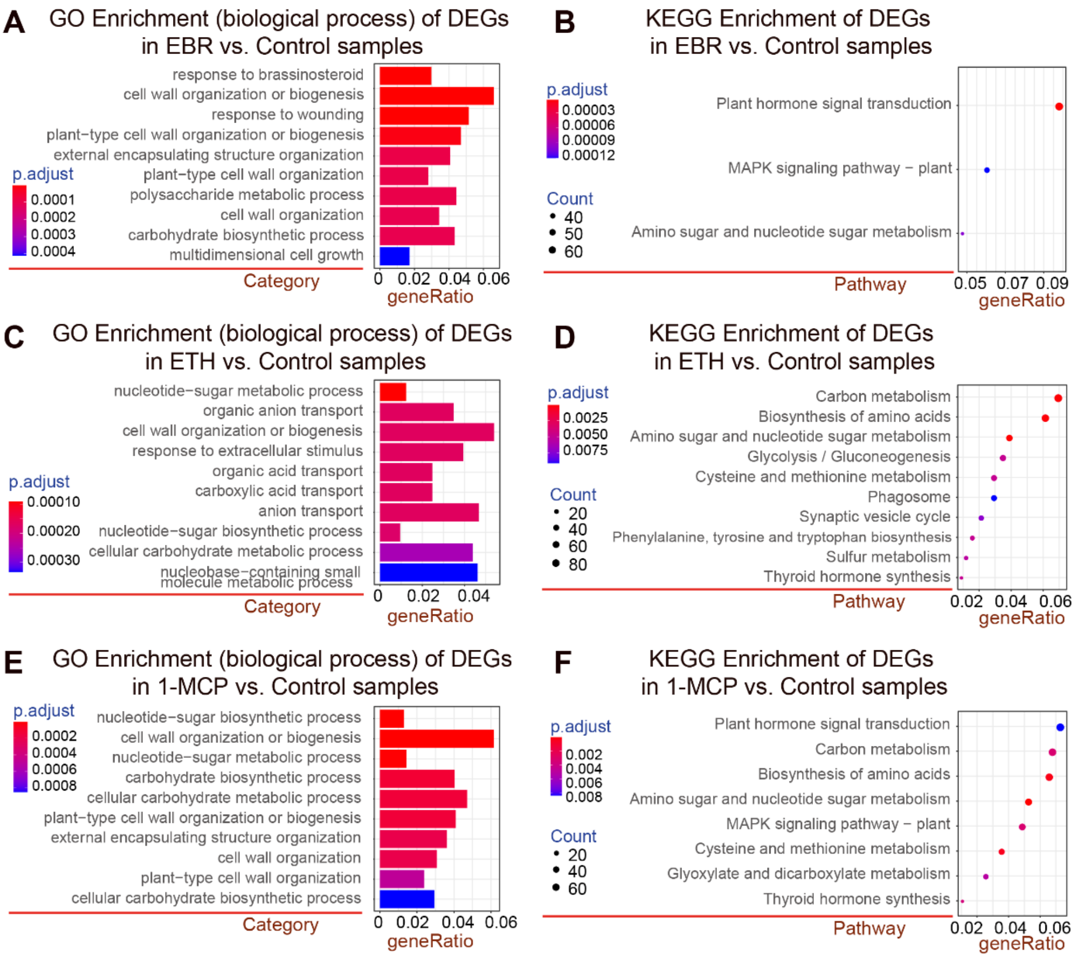

Supplementary Figure S2. GO and KEGG enrichment analyses were performed on the RNA-seq under different treatments. (A, C, and E) GO enrichment analysis of differentially expressed genes (DEGs) in EBR, ETH, and 1-MCP treatments compared to the control. (B, D, and F) KEGG enrichment analysis revealing the enriched pathways of DEGs in EBR, ETH, and 1-MCP treatments compared to the control. GO, Gene ontology. KEGG, Kyoto encyclopedia of genes and genomes.

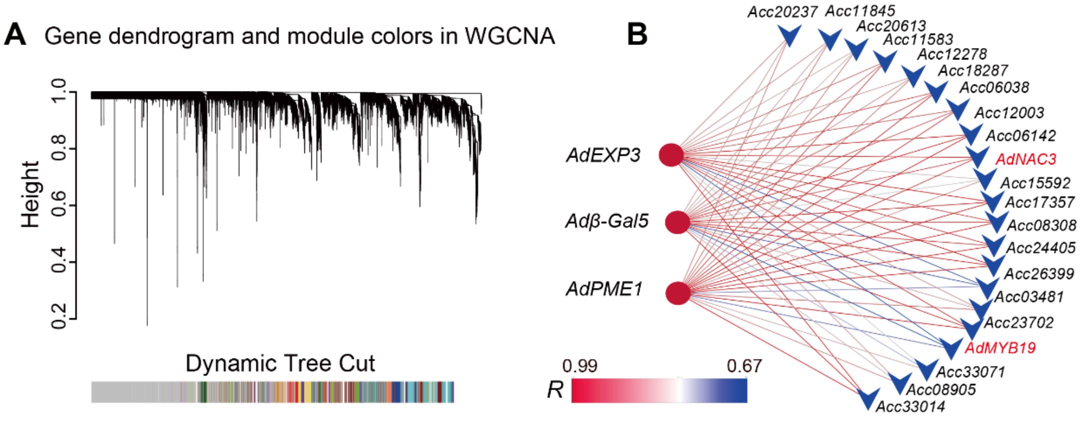

Supplementary Figure S3. Clustering and correlation analyses based on transcriptome data. (A) WGCNA analysis reveals the cluster dendrogram among different samples. (B) Correlation analysis of 21 selected TFs with three cell wall-related genes. Lines between different connections represent *Pearson* correlation coefficients.

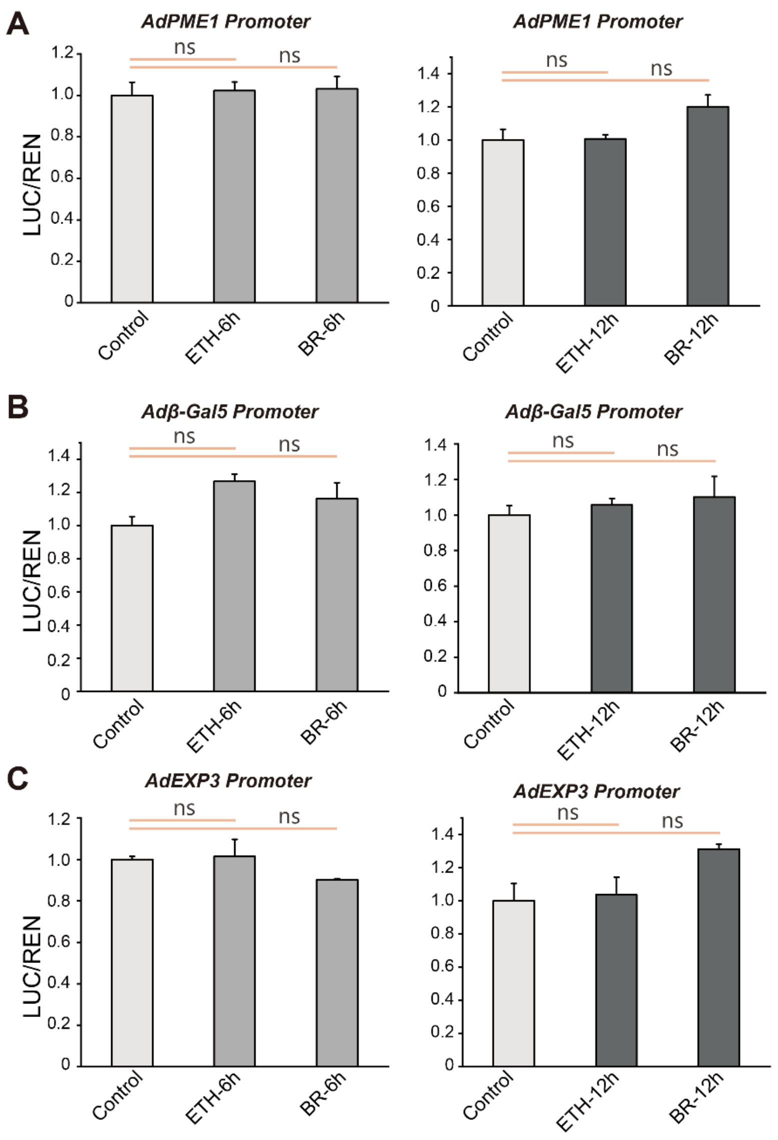

Supplementary Figure S4. Promoter activity analysis of functional genes. Validation was conducted in a *Nicotiana benthamiana* system under ethylene-generating compound ethephon (ETH) and brassinosteroid (BR) analog 2,4-epibrassinolide treatments for 6 and 12 hours to investigate their effects on the promoters of (A) *AdPME1*, (B) *Adβ-Gal5*, and (C) *AdEXP3*. The LUC/REN value of control was set as 1. Error bars represent SE based on 4 biological replicates. Statistical significance was determined using Student's t-test. ns indicates no significant difference ($P \geq 0.05$).

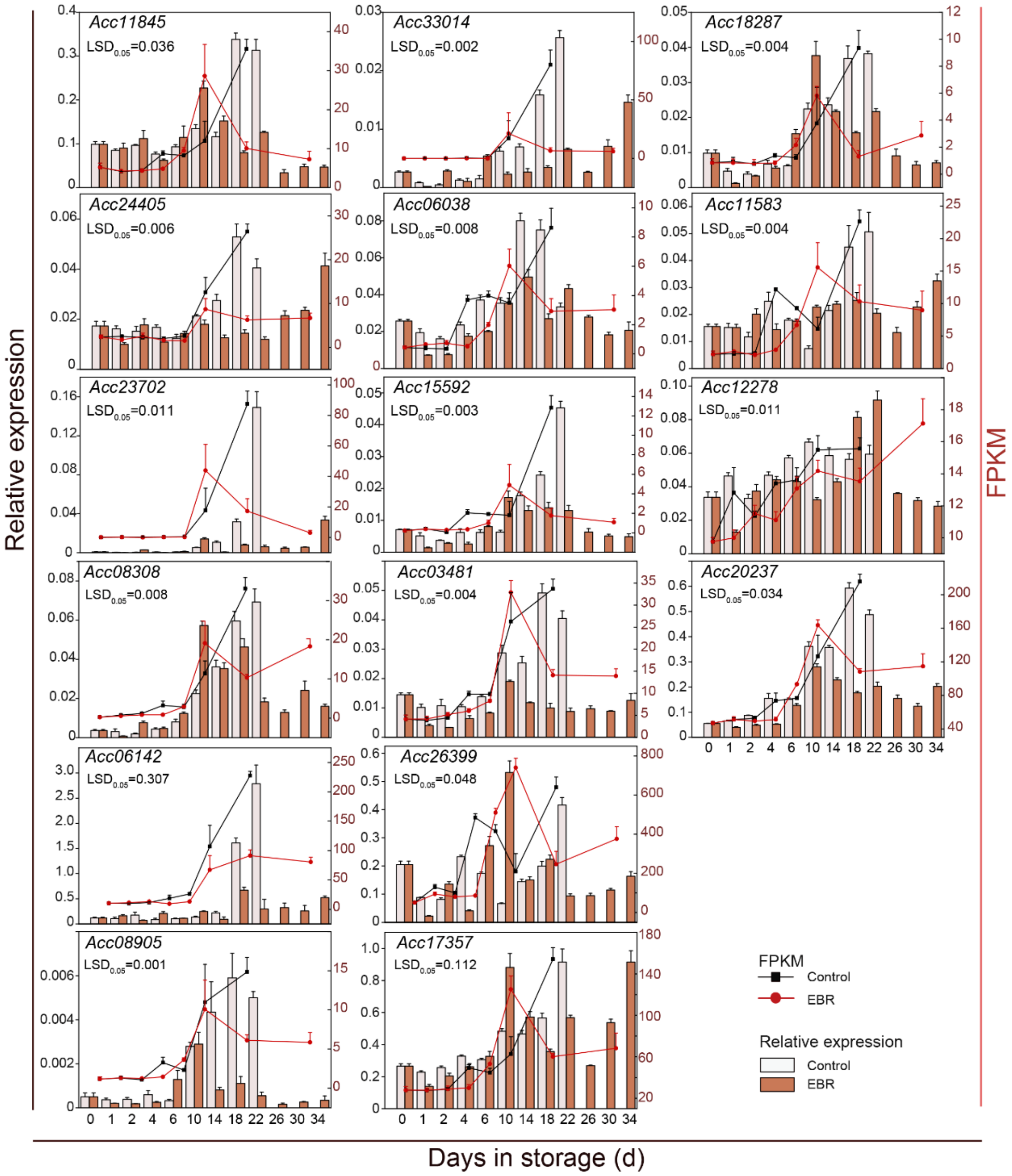

Supplementary Figure S5. Expression levels of other TF genes during the ripening process of 'Hayward ' fruits following EBR treatment. EBR, brassinosteroid analog 2,4-epibrassinolide. FPKM (fragments per kilobase of transcript per million mapped reads), a normalized unit for gene expression in transcriptome analysis. Error bars represent SE based on 3 biological replicates, and LSD indicates significant differences by least significant difference method (*P* = 0.05).

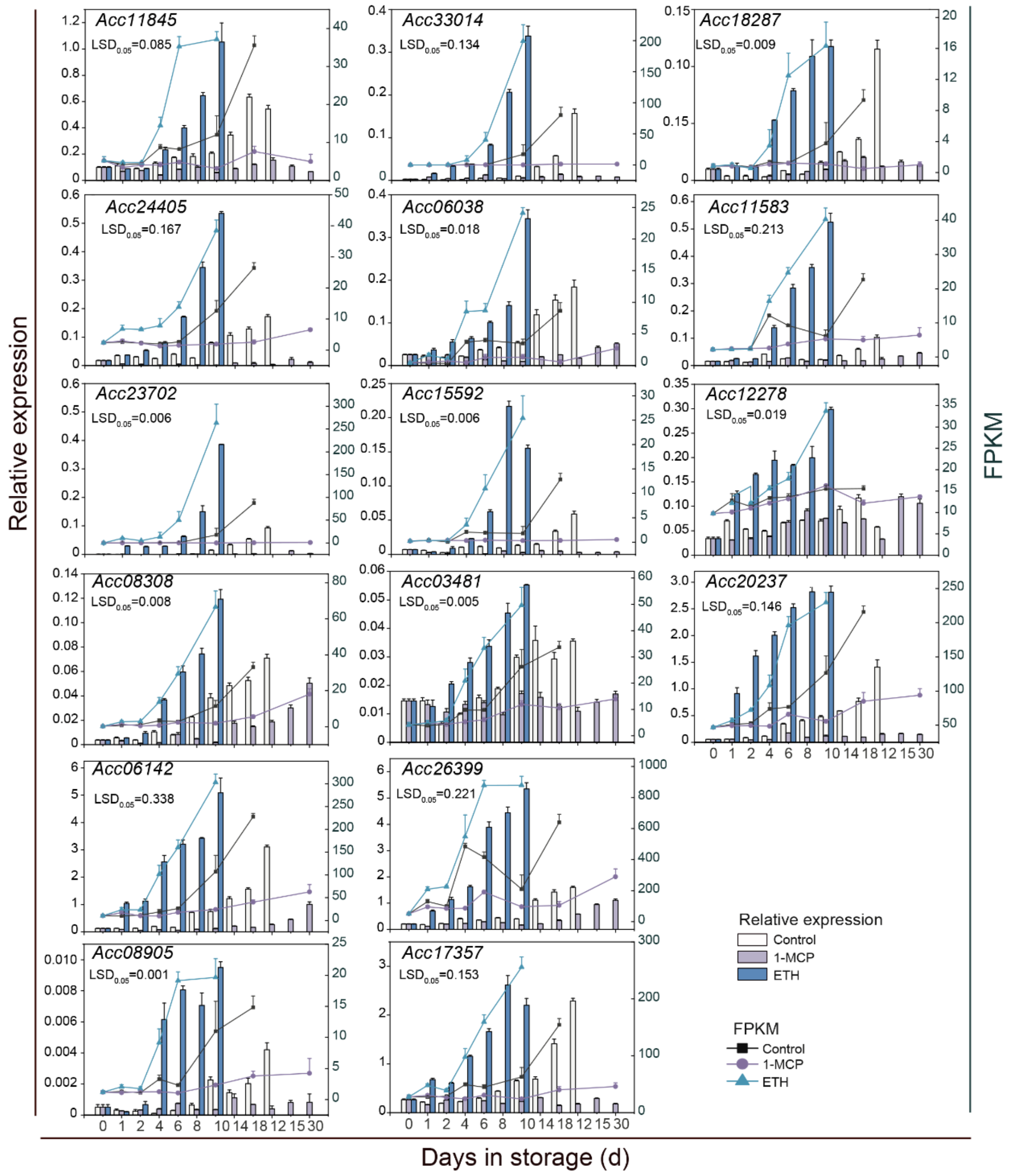

Supplementary Figure S6. Expression levels of other TF genes during the ripening process of 'Hayward ' fruits following ETH treatment. ETH, ethylene. 1-MCP, 1-methylcyclopropene, an ethylene receptor antagonist. FPKM (fragments per kilobase of transcript per million mapped reads), a normalized unit for gene expression in transcriptome analysis. Error bars represent SE based on 3 biological replicates, and LSD indicates significant differences by least significant difference method (P = 0.05).

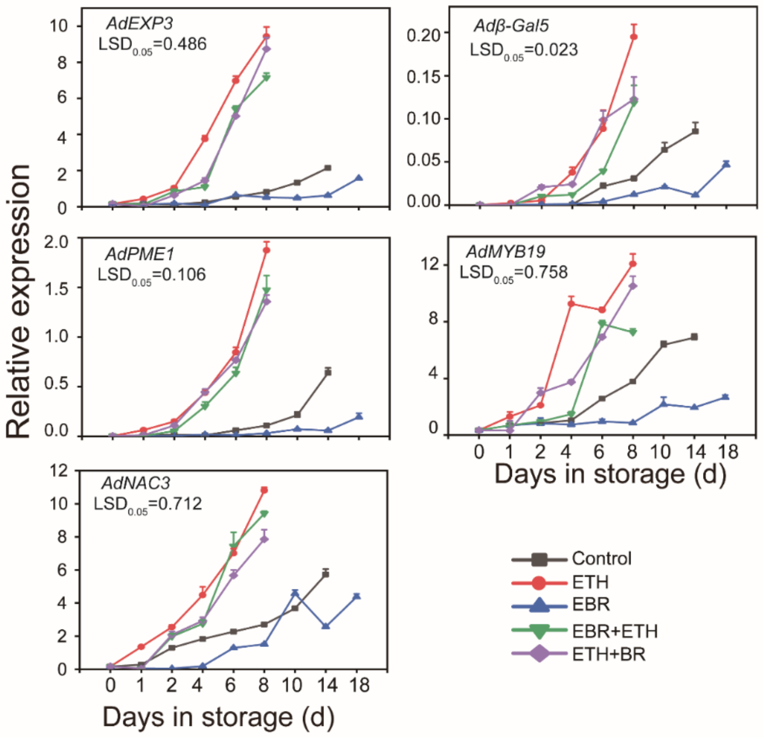

Supplementary Figure S7. Expression levels of *AdEXP3*, *Adβ-Gal5*,*AdPME1*, *AdMYB19* and *AdNAC3* during the ripening process of 'Hayward' fruits following individual treatments of ETH and EBR, as well as the combined treatment of ETH and EBR(EBR+ETH and ETH+EBR). EBR, brassinosteroid analog 2,4-epibrassinolide. ETH, ethylene. Error bars represent SE based on 3 biological replicates, and LSD indicates significant differences by least significant difference method (P = 0.05).

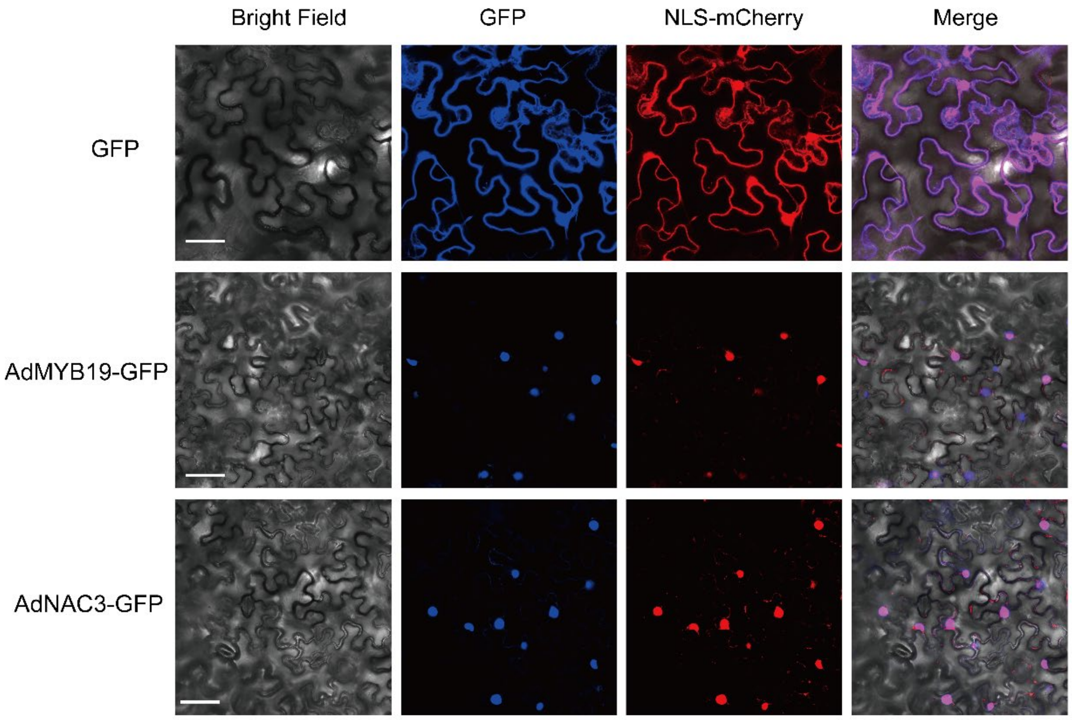

Supplementary Figure S8. Subcellular localization analysis of AdMYB19 and AdNAC3.GFP, green fluorescent protein. NLS-mCherry serves as a nuclear localization control, with a scale bar of 50 μm.

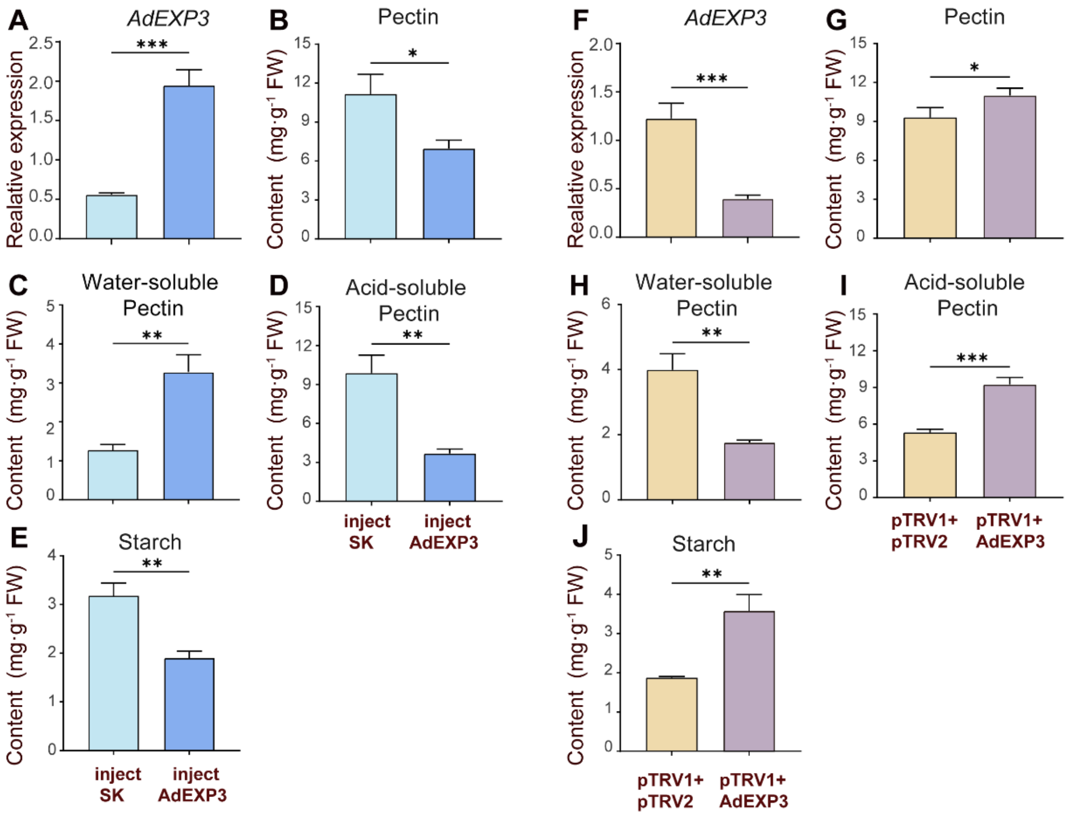

Supplementary Figure S9. Verification of the function of the AdEXP3 in 'Hayward' kiwifruit. (A-E) Transient overexpression of *AdEXP3* in kiwifruit was carried out to measure the expression level of *AdEXP3*, pectin content, water-soluble pectin content, acid-soluble pectin content and starch content. (F-J) Transient silencing of *AdEXP3* was performed to measure the softening indicators of the fruit. SK and pTRV1+ pTRV2 represents the empty vector(control). Error bars represent SE based on 3 biological replicates. Asterisks indicate significant differences in Student's t test (*P < 0.05, **P < 0.01; ***P < 0.001), FW, fresh weight of the fruit.

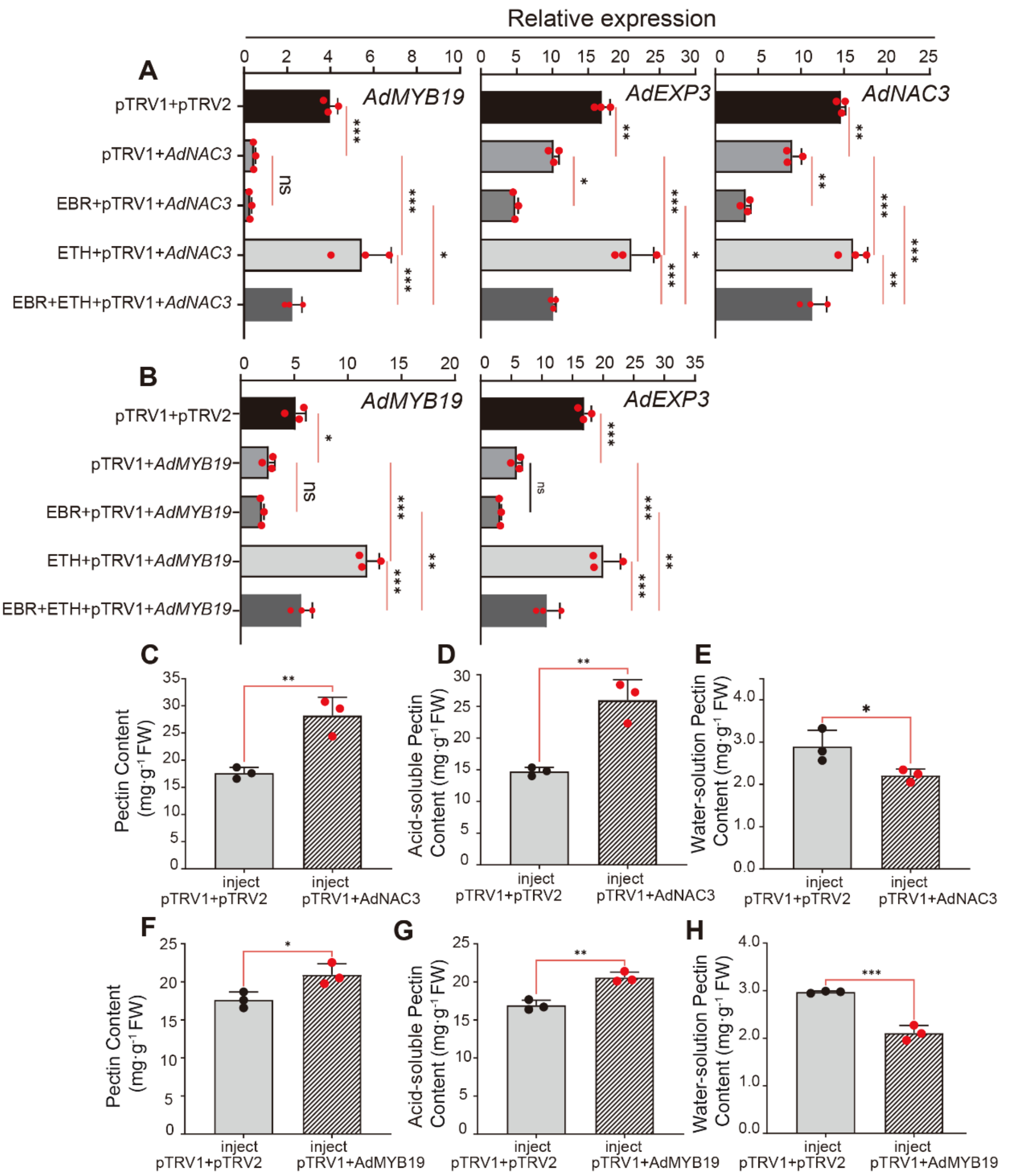

Supplementary Figure S10. Transient silencing validation of AdNAC3 and AdMYB19 functions in 'Hayward ' fruits. (A-B) Transient silencing expression of *AdNAC3* and *AdMYB19* in kiwifruit. RT-qPCR validation of *AdNAC3*, *AdMYB19*, and *AdEXP3* mRNA levels under EBR treatment, ETH, and combined EBR and ETH treatments. (C-H) Determination of changes in fruit softening indicators, pectin, acid-soluble pectin, and water-soluble pectin content following transient silencing of *AdNAC3* and *AdMYB19* genes. Error bars represent SE based on 3 biological replicates. pTRV1+ pTRV2 represents an empty vector(control), and asterisks indicate significant differences by Student's t-test (*$P < 0.05$, **$P < 0.01$; ***$P < 0.001$).

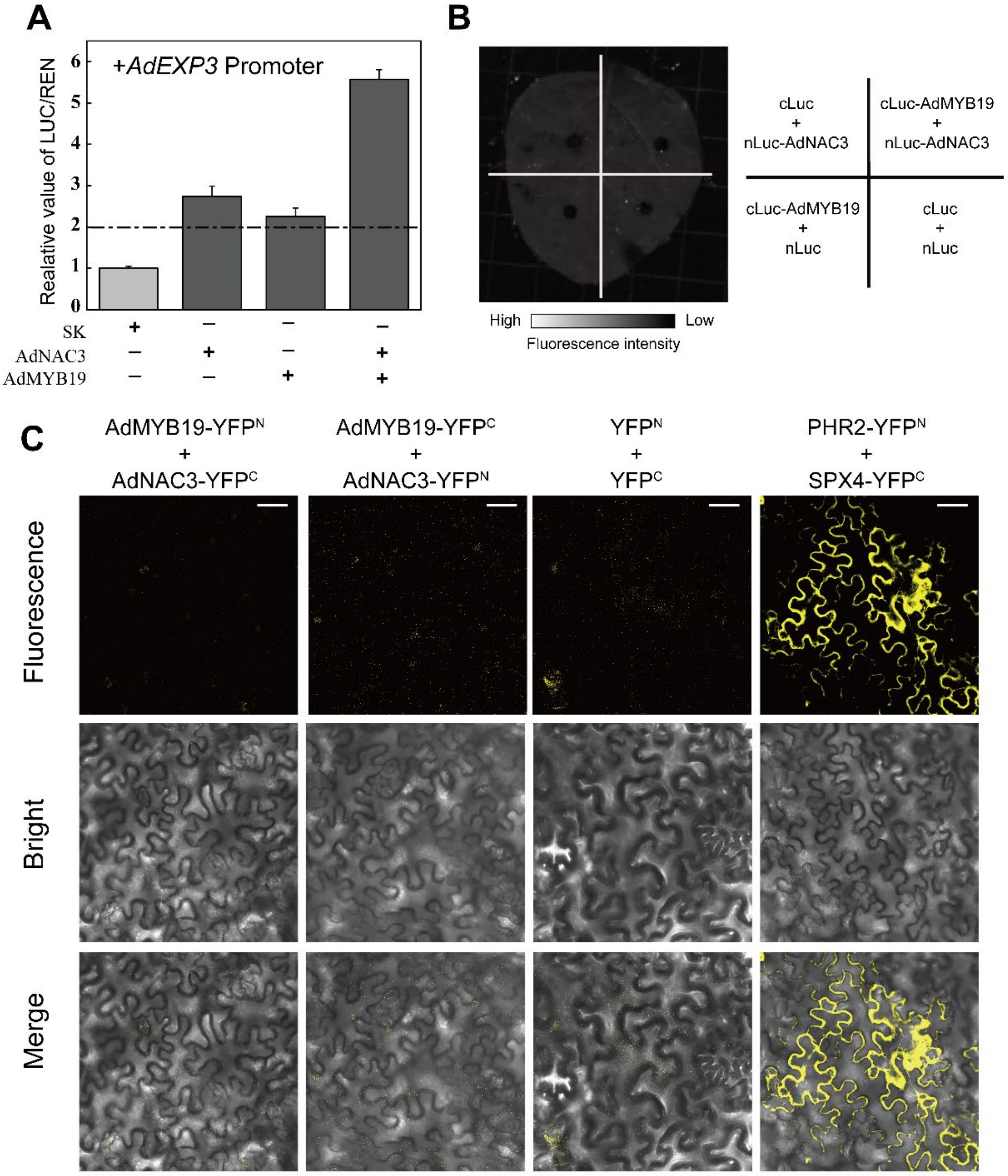

Supplementary Figure S11. AdNAC3 and AdMYB19 do not physically interact at the protein level. (A) Dual-luciferase assay to validate the cooperative regulatory effect of AdNAC3 and AdMYB19 on the *AdEXP3* gene. Error bars represent SE based on 4 biological replicates. (B) Firefly luciferase complementation imaging (LCI) experiment results indicate that there is no protein interaction between AdNAC3 and AdMYB19. nLuc-AdNAC3 and cLuc-AdMYB19 fusion constructs were co-transformed into *Nicotiana benthamiana*, with cLuc+ nLuc-AdNAC3, cLuc-AdMYB19 + nLuc, and nLuc + cLuc used as negative controls. (C) Bimolecular fluorescence complementation (BiFC) experiment further confirms the absence of protein interaction between AdNAC3 and AdMYB19. The N-terminal and C-terminal YFP fragments (represented as YFP^N and YFP^C) were fused with the C-terminal and N-terminal of AdNAC3 and AdMYB19, respectively. YFP^N and YFP^C constructs vectors were used as negative controls.

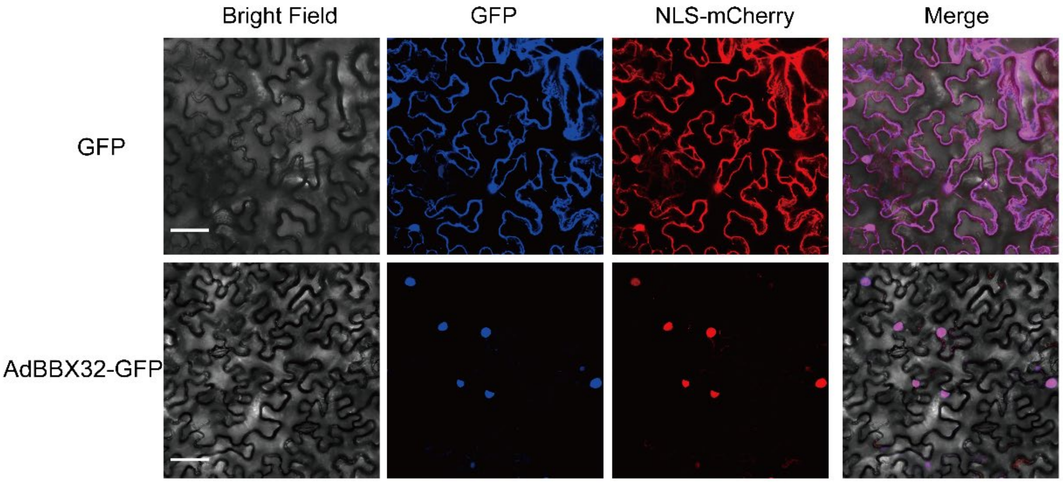

Supplementary Figure S12. Subcellular localization analysis of TF AdBBX32. GFP, green fluorescent protein. NLS-mCherry serves as a nuclear localization control, with a scale bar of 50μm.

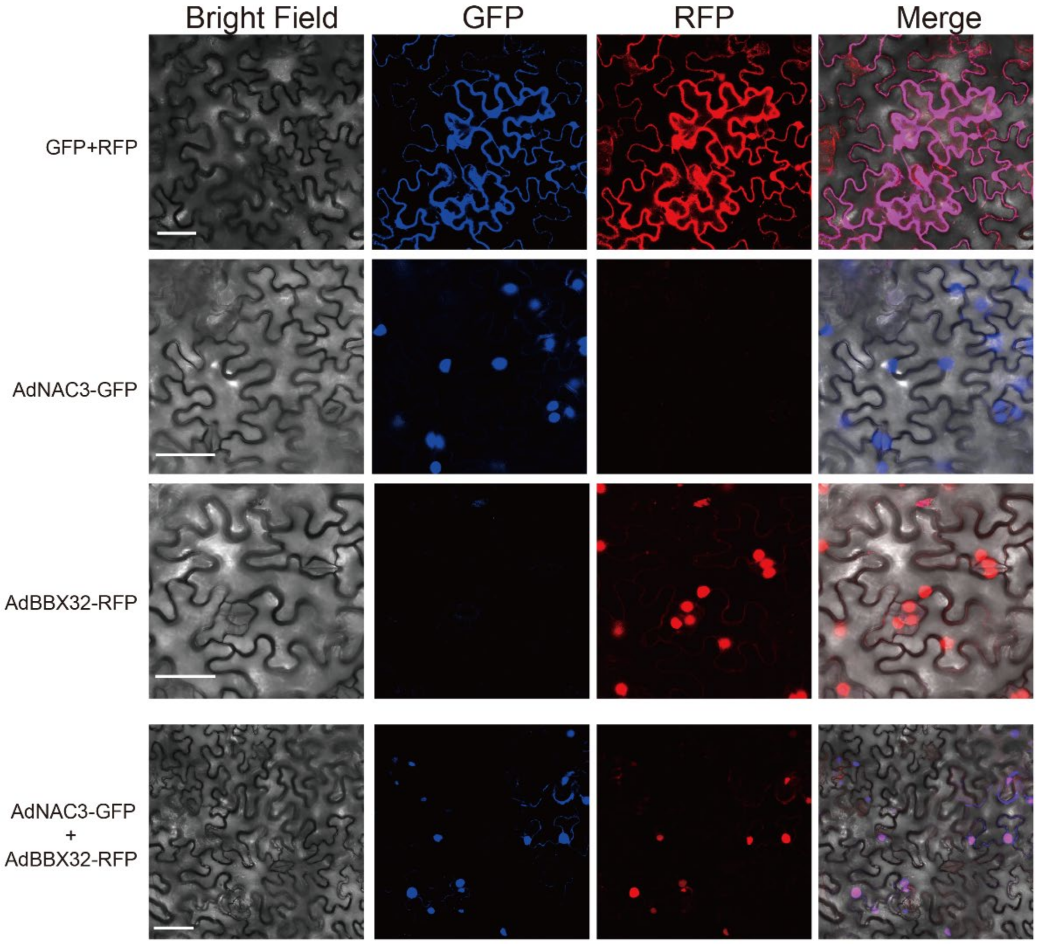

Supplementary Figure S13. Subcellular colocalization analysis of AdNAC3 and AdBBX32. GFP, green fluorescent protein. RFP, red fluorescent protein. Scale bar is 50 μm.

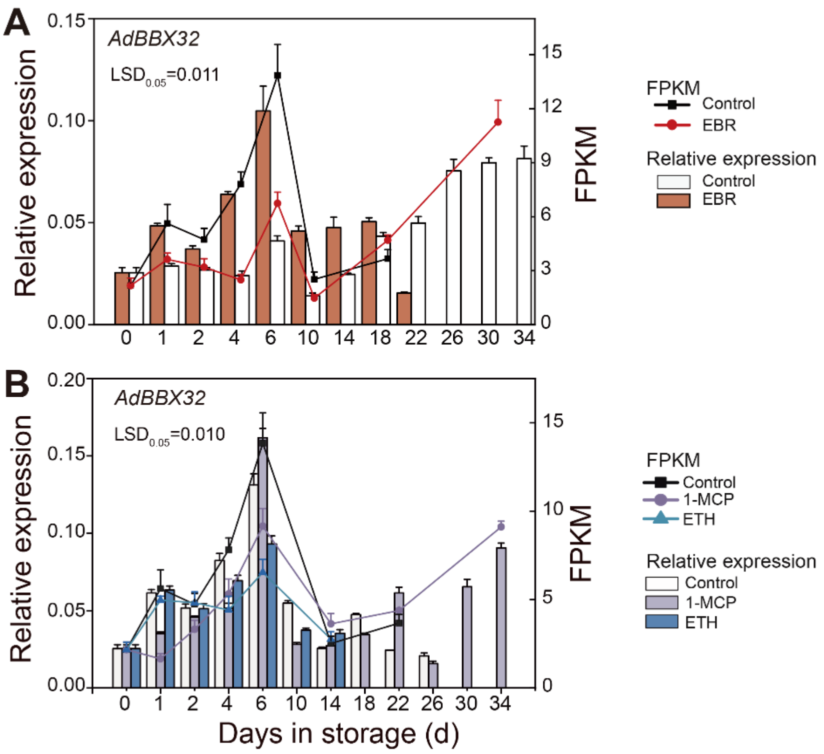

Supplementary Figure S14. Expression levels of *AdBBX32* under ETH and EBR treatment conditions. EBR, brassinosteroid analog 2,4-epibrassinolide. ETH, ethylene. 1-MCP, 1-methylcyclopropene, an ethylene receptor antagonist. Error bars represent SE based on 3 biological replicates. FPKM (fragments per kilobase of transcript per million mapped reads), a normalized unit for gene expression in transcriptome analysis. LSD indicates significant differences by least significant difference method ($P = 0.05$).

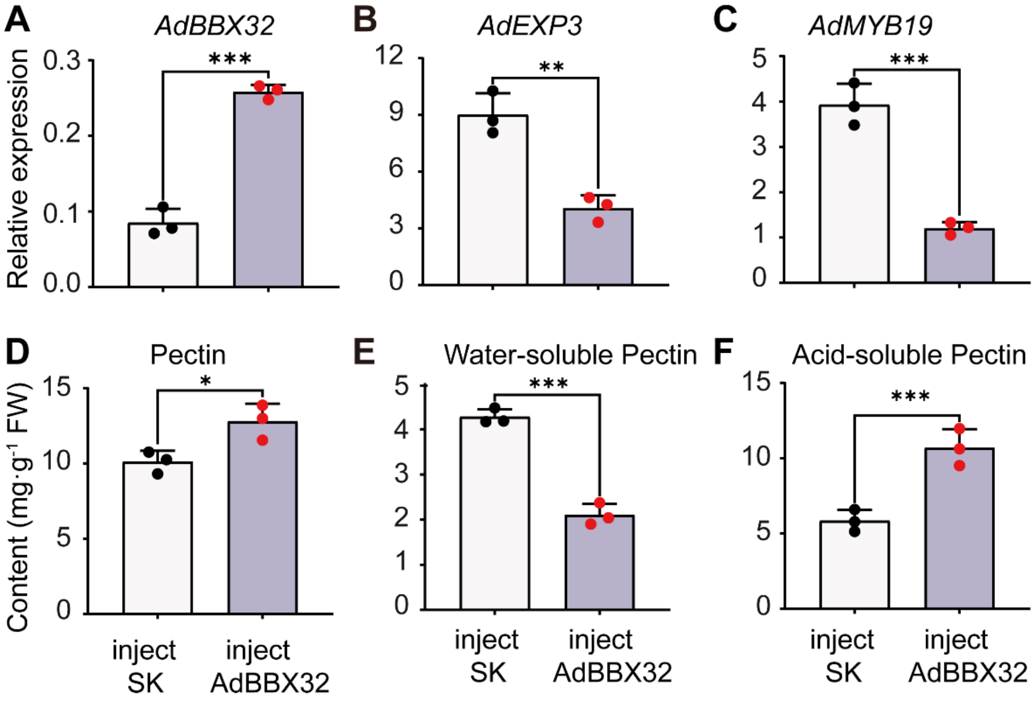

Supplementary Figure S15. Transient overexpression validation of AdBBX32 function in 'Hayward' kiwifruit. RT-qPCR verification of (A) *AdBBX32*, (B) *AdEXP3* and (C) *AdMYB19* transcription levels on 3 d after transient overexpression of *AdBBX32*. Analysis of changes in fruit softening indicators, such as (D) pectin, (E) water-soluble pectin, and (F) acid-soluble pectin content, due to transient overexpression of *AdBBX32*. FW, fresh weight of the fruit. SK represents the empty vector, and asterisks indicate significant differences by Student's t-test (**P*<0.05, ***P*<0.01; ****P*<0.001).

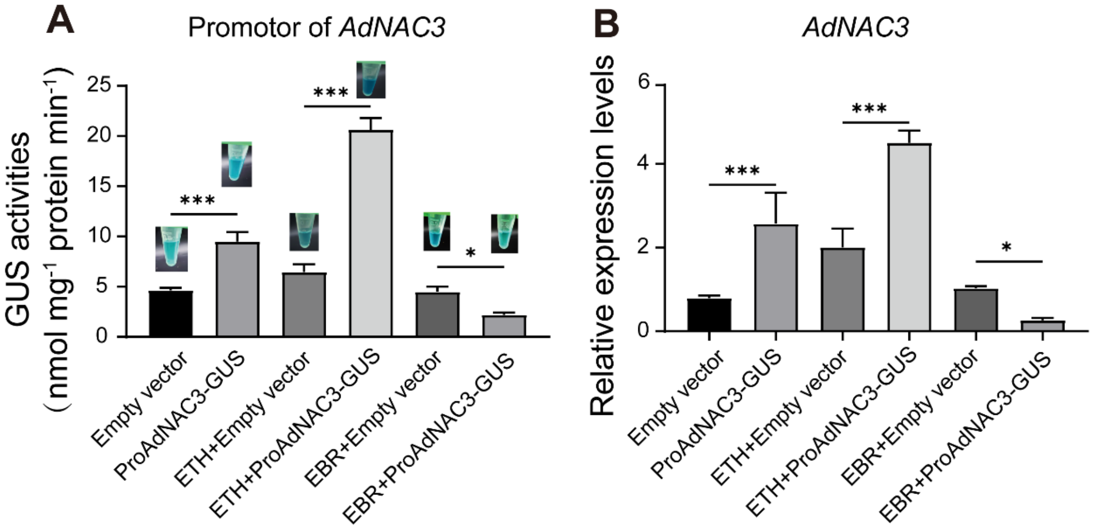

Supplementary Figure S16. Transient overexpression of the *AdNAC3* promoter in kiwifruit to validate its promoter activity. (A) GUS staining and GUS enzyme activity assays further evaluated the response of the *AdNAC3* promoter (ProAdNAC3) to ETH and EBR, and (B) RT-qPCR was used to analyze the expression pattern of *AdNAC3*. EBR, brassinosteroid analog 2,4-epibrassinolide. ETH, ethylene. Error bars represent SE based on 3 biological replicates. Asterisks indicate significant differences by Student's t-test (**P*<0.05, ***P*<0.01; ****P*<0.001).

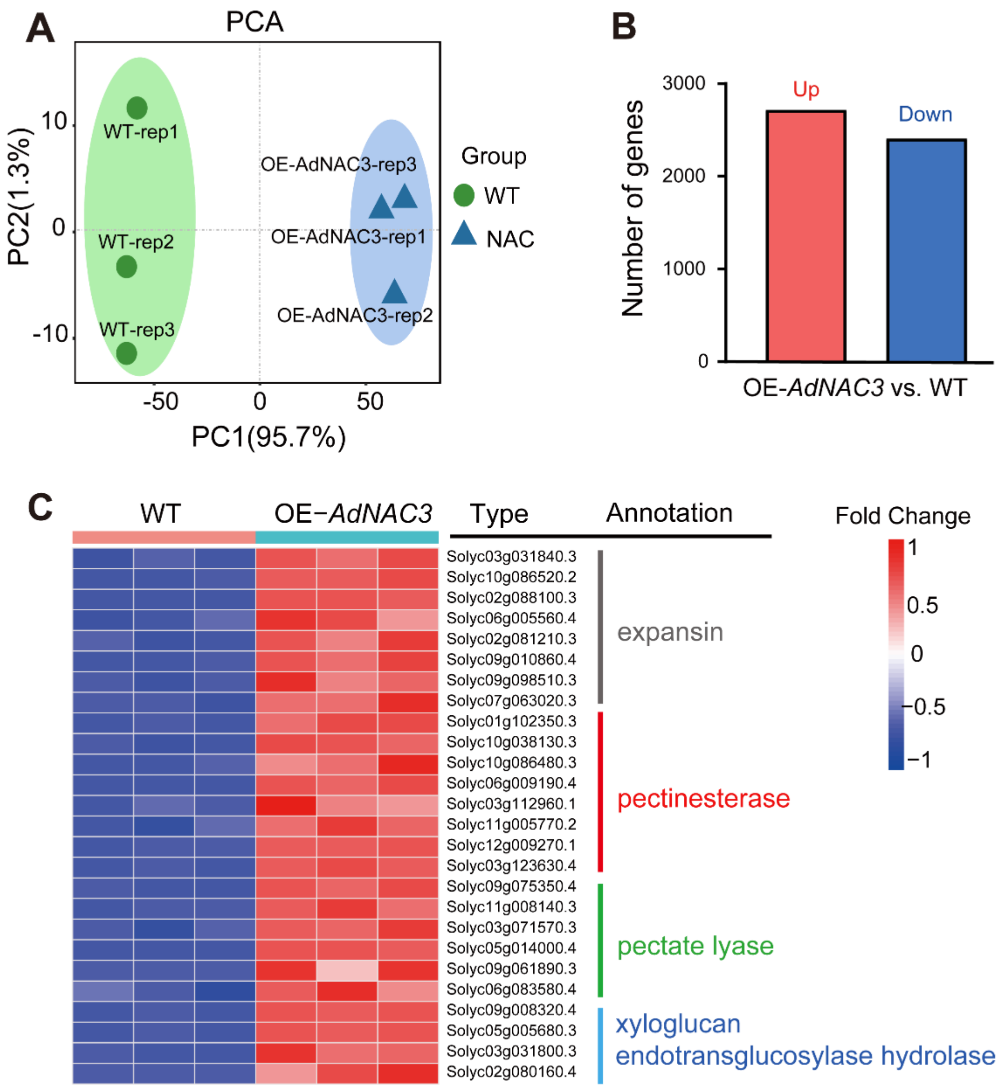

Supplementary Figure S17. RNA-seq analysis on the 7d break stage (Br+7) revealed the molecular basis of fruit ripening in the stable overexpression 'Micro-Tom' transgenic lines of *AdNAC3*. (A) Principal component analysis (PCA) analysis of wild-type (WT) and overexpression *AdNAC3*(OE-*AdNAC3*) lines. rep, replicate. (B) Number of differentially expressed genes (DEGs) from RNA-seq in OE-*AdNAC3* and WT. (C) Expression profiles of 26 selected cell wall-related DEGs. The color scale represents the Z-score normalized values. The values range from -1 to 1, and the scale is labeled as 'Fold Change'.
